# Separator Engineering for Sodium Metal Batteries: Challenges, Rational Design and Recent Modification Strategies

**DOI:** 10.1002/adma.202510480

**Published:** 2025-08-15

**Authors:** Lichun Wang, Zhenzhen Wang, Yun Bai, Ding Yuan, Jun Chen, Shi‐Xue Dou, Hua‐Kun Liu, Chao Wu

**Affiliations:** ^1^ School of Chemical Engineering and Materials Changzhou Institute of Technology Changzhou 213022 P. R. China; ^2^ Institute of Energy Materials Science University of Shanghai for Science and Technology Shanghai 200093 P. R. China; ^3^ Institute for Superconducting and Electronic Materials Australian Institute for Innovative Materials University of Wollongong New South Wales 2525 Australia

**Keywords:** dendrite suppression, electrochemical evaluation, Na metal anodes, separator engineering

## Abstract

Sodium metal batteries (SMBs) have emerged as promising candidates for next‐generation energy storage systems, leveraging their high theoretical capacity and the natural abundance of Na resources. Nevertheless, critical challenges, including dendritic growth, side reactions, and pronounced volume fluctuations during cycling, continue to impede their commercialization. Conventional separators, including polyolefin or glass fiber types, suffer from poor wettability, uneven ion flux, and a rough surface. To tackle these challenges, functional separator engineering encompassing interfacial chemistry regulation, multiscale structural design, and hybrid integration of advanced materials has demonstrated remarkable progress in regulating Na⁺ flux, stabilizing SEI, and suppressing dendrite propagation. This review systematically provides a comprehensive summary of recent developments in separator engineering for SMBs, including modified polyolefin separators, enhanced glass fiber frameworks, cellulose‐based separators, MOFs/COFs‐based, and emerging separators. In addition, a comprehensive electrochemical evaluation framework encompassing CE in half‐cells is proposed, the lifespan of symmetric cells, full cell performance, and safety validation to assess practical applicability. Furthermore, computational simulations for mechanism elucidation and predictive design, as well as future separator perspectives, have been also discussed to guide the development of next‐generation separators for high‐performance and commercially viable SMBs.

## Graphical abstract

This review covers advances in sodium metal battery separators, focusing on interfacial stabilization, dendrite suppression, and ion transport. It analyzes diverse materials, proposes comprehensive evaluation methods, and discusses simulations and future directions for high‐performance, safe, and practical separators.

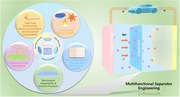

## 1 Introduction

With the intensification of climate change and the continuous depletion of fossil fuel resources, the global energy structure is undergoing a rapid transition toward renewable sources. To address the intrinsic intermittency of solar and wind energy, the development of efficient and reliable energy storage systems has become essential. Meanwhile, the fast‐growing deployment of electric vehicles (EVs) imposes increasingly stringent requirements on decarbonizing the transportation sector. Under this dual impetus, the development of advanced battery technologies with high energy density, low cost, and excellent safety is regarded as a key enabler for achieving sustainable development.^[^ ^1^, ^2^, ^3^, ^4^, ^5^ ^]^ Although lithium‐ion batteries (LIBs) currently dominate the market, the limited availability and uneven geographical distribution of lithium resources have triggered rising material costs and increasing supply chain risks, thereby prompting the exploration of alternative energy storage chemistries based on more abundant elements.

In this context, sodium‐ion batteries (SIBs) have attracted growing attention as a promising next‐generation electrochemical energy storage system, owing to their structural and operational similarity to LIBs, as well as the abundance and low cost of sodium resources. These features make SIBs particularly suitable for large‐scale energy storage applications where cost‐effectiveness is critical. However, the relatively large ionic radius of Na^+^ and its lower insertion potential result in limited energy and power densities, making SIBs less competitive in energy‐intensive scenarios such as electric transportation. To overcome these limitations, much research effort has been devoted to developing high‐energy metal‐based battery configurations. Among them, SMBs, featuring ultra‐high theoretical capacity (1166 mAh g^−1^) and extremely low redox potential (−2.71 V vs SHE), are regarded as one of the most promising candidates to break through the energy density bottleneck.^[^ ^6^, ^7^, ^8^ ^]^

Such advantages enable SMBs to potentially offer a sustainable and economically viable pathway to meet the escalating demand for large‐scale energy storage, particularly for renewable grid integration and electric vehicles.^[^ ^9^, ^10^ ^]^ However, the practical application of SMBs is severely hampered by uncontrollable dendritic Na growth, unstable solid electrolyte interface (SEI) layers, severe volume changes during cycling, and persistent parasitic reactions, which collectively lead to shorter cycle life and reduced safety.^[^ ^11^, ^12^ ^]^

These challenges stem primarily from the sodium metal anode (SMA), where high reactivity induces interfacial instability and accelerates dendrite growth. Unlike conventional LIBs and SIBs, where Li and Na is stored within the crystal structure of the anode materials, SMBs require more stringent requirements on ion flux uniformity, volume change, and interfacial stability.^[^ ^13^, ^14^, ^15^ ^]^ In this context, the separator, which has traditionally acted as a passive physical barrier between electrodes, has evolved into an active functional platform that controls ion transport pathways, stabilises interfacial reactions, and inhibits dendritic propagation.^[^ ^16^, ^17^ ^]^ Serving as the primary conduit for ionic transport, an electronic insulator, and a reservoir for electrolyte, the separator significantly impacts overall battery performance and safety. Its micro and nanostructural features, including porosity, pore size distribution, tortuosity, and thickness, along with mechanical properties such as modulus and toughness, chemical and electrochemical stability, and surface physicochemical characteristics, profoundly influence several key aspects: i) the uniformity of Na^+^ flux, which is a determining factor for suppressing dendrite nucleation and growth; ii) the local chemical environment at the electrode–electrolyte interface and the formation and evolution of the solid electrolyte interphase (SEI), which are vital to interfacial stability; iii) the transmission of internal mechanical stress and the accommodation of volumetric changes during cycling; iv) the overall safety of the battery, including resistance to dendrite penetration and thermal stability.

However, conventional commercial separators, such as polyolefin‐based (PP/PE) separators, exhibit serious limitations when applied to SMBs.^[^ ^18^ ^]^ They exhibit poor wettability toward ester‐based electrolytes, resulting in low CE of Na plating/stripping. The unoptimized pore structure tends to induce uneven Na + flux, which exacerbates dendrite growth. In addition, their limited chemical stability and poor tolerance to the highly reducing nature of Na metal raise serious concerns for long‐term interfacial compatibility. Moreover, the flexibility and thermal dimensional stability of these separators often fall short under the stringent requirements of extended cycling and wide‐temperature operation in SMBs.^[^ ^19^ ^]^ Although glass fibre (GF) separators offer enhanced electrolyte absorption and wettability, their high porosity and random fibre distribution tend to cause local current density fluctuations, further promoting infiltration of dendritic growths and undesirable side effects.^[^ ^20^ ^]^ These limitations underscore the necessity of developing separators with tailored properties to meet the specific demands of SMB systems. As a result, the design of high‐performance separators optimized for Na metal chemistry has emerged as a critical enabler for the practical realization of this promising battery technology.

Recent advances in separator design have introduced innovative approaches to address these issues. Strategies such as interfacial chemical modulation, multiscale structural engineering, and integration of advanced hybrid organic–inorganic materials have shown improvements in electrolyte wettability, mechanical strength, thermal stability, and ion selectivity.^[^ ^21^ ^]^ Advanced separators incorporating materials such as metal‐organic frameworks (MOFs),^[^ ^22^ ^]^ covalent organic frameworks (COFs),^[^ ^23^ ^]^ cellulose‐based networks, and Janus architectures have demonstrated great potential to regulate Na^+^ ions flux, promote robust SEI formation, and effectively mitigate dendritic evolution.

This review provides the first comprehensive and systematic summary of recent advances in separator engineering for SMBs, with a particular focus on dendrite and side reactions suppression, interfacial stabilization, and ion transport optimization. To provide a holistic understanding of separator engineering in SMBs, this review is structured as follows. We begin by elucidating the fundamental challenges of SMA, including parasitic side reactions, unstable SEIs, dendritic growth, and volume fluctuations and their underlying physicochemical mechanisms. We then critically examine the limitations of conventional commercial separators, emphasizing their incompatibility with sodium electrochemistry. Building on these insights, we define the distinct functional requirements for separators in SMBs, which encompass ionic conductivity, Na⁺ flux regulation, mechanical adaptability, and interfacial compatibility. The core of this review focuses on recent advances in separator modification strategies, spanning interfacial chemical functionalization, multiscale structural engineering, and advanced composite designs across a range of material systems, including polyolefins, GF, cellulose‐based separators, and MOF/COF‐derived architectures. We further introduce a multidimensional electrochemical evaluation framework for assessing separator performance under practical conditions, and discuss the emerging role of computational modeling and operando characterization in guiding separator design. Finally, we outline future research directions and interdisciplinary opportunities to accelerate the development of safe, high‐performance, and scalable SMB technologies.

## 2 Key challenges for SMAs

**Figure 1:**
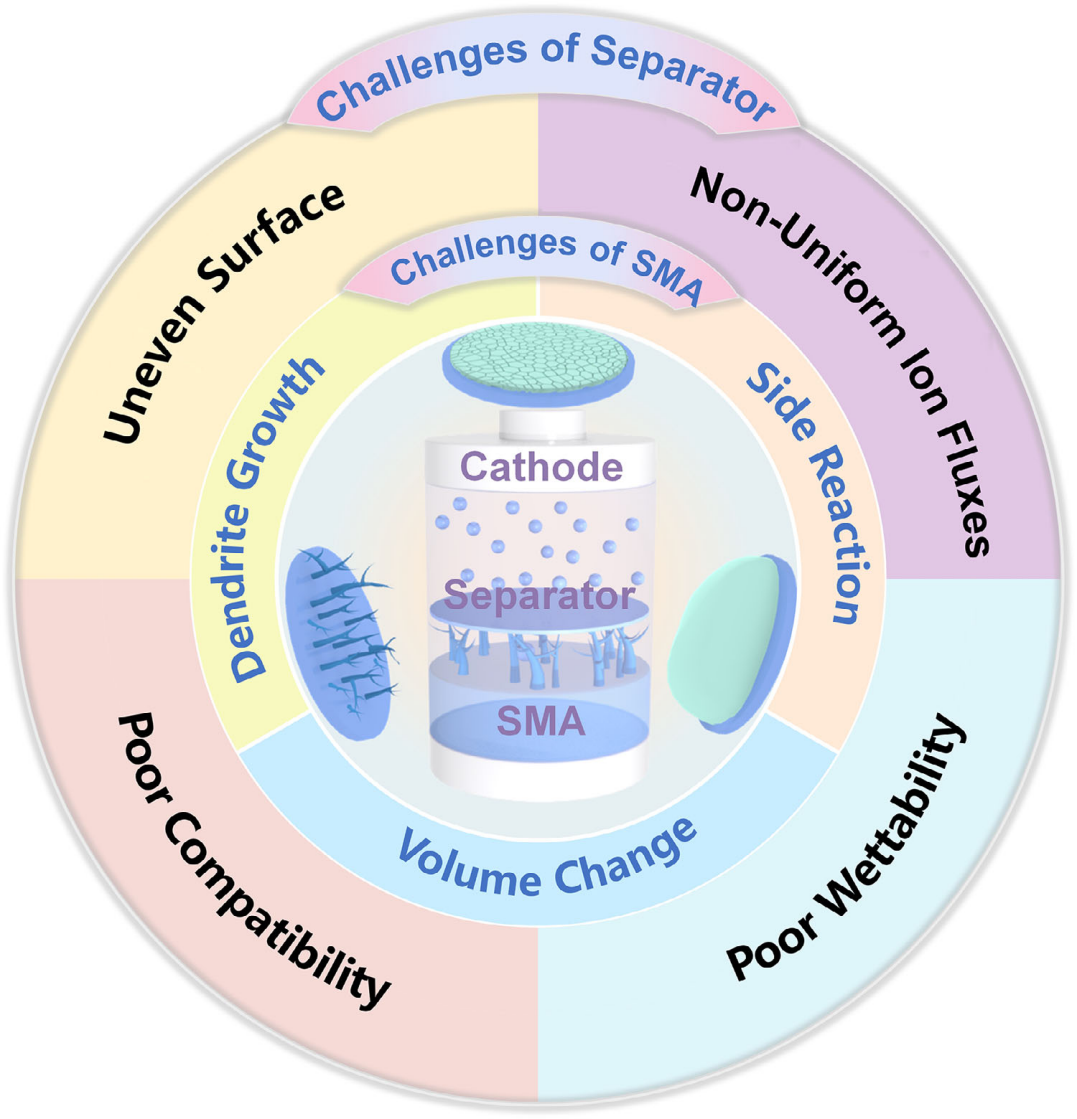
Key challenges of SMA and Separator.

Due to the high reactivity and low electrochemical potential of Na metal, SMBs face formidable challenges. Issues such as serious side reactions, unstable SEI, dendritic growth, and volume fluctuations significantly compromise cycling stability, reversibility, and safety (**Figure** **1**).^[^ ^24^, ^25^ ^]^ This section systematically discusses these challenges and elucidates the fundamental mechanisms of Na^+^ deposition.

### 2.1 The Side Reactions and the Unstable SEI

**Figure 2:**
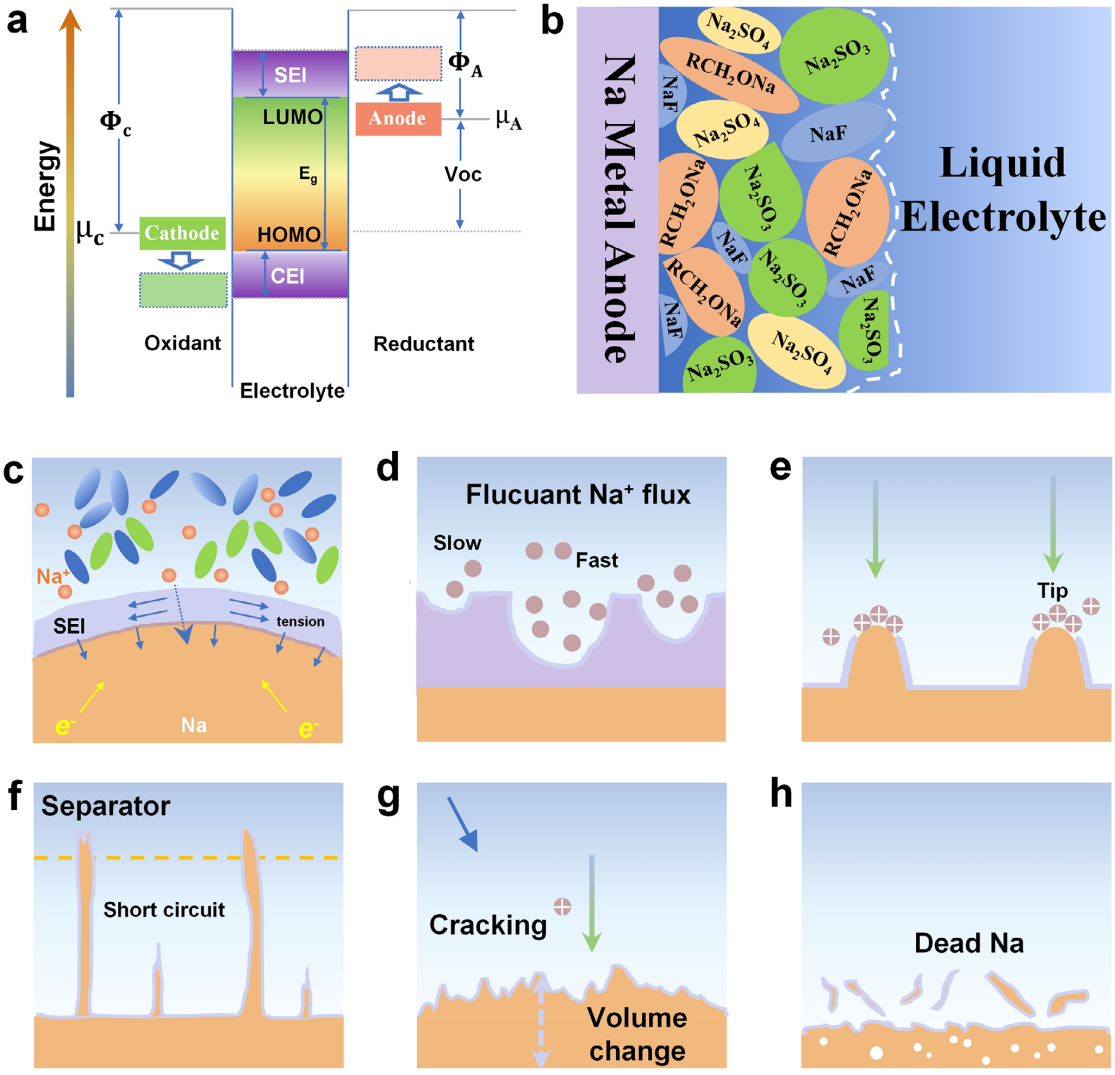
Schematic representation of the interfacial evolution and failure mechanism of SMA in liquid electrolyte. a,b) Schematic illustration of SEI structures formed at the Na‐liquid electrolyte interface. c) Tension generated by Na^+^ across the SEI layer. d) Na^+^ deposition on an inhomogeneous SEI layer. e) Schematic of the growth of Na dendrites. f) Schematic representation of the anomalous growth of dendrites penetrating a spacer. g) Illustration of SMA volume expansion. h) Electroplating produces a “dead Na” process. Reproduced with permission.^[^ ^34^ ^]^ Copyright 2023, The Royal Society of Chemistry.

Since the SEI was first reported on the anode surface in the 1970s, it has remained a central focus in the study of non‐aqueous rechargeable batteries.^[^ ^26^, ^27^ ^]^ However, in the context of SMBs, the stability of the SEI is strongly influenced by the interfacial reactions between the electrolyte and Na anode.^[^ ^28^, ^29^ ^]^ The side reactions often lead to the formation of unstable SEI layers, which in turn exacerbate dendrite growth and shorten battery lifespan.^[^ ^30^, ^31^ ^]^ **Figure** **2**a illustrates the energy landscape between the electrode and the electrolyte.^[^ ^32^, ^33^ ^]^ In an ideal thermodynamically stable system, the chemical potentials of the anode (µA) and cathode (µC) should reside within the electrochemical stability window of the electrolyte (Eg), thereby avoiding interfacial side reactions. However, in practical scenarios, both µA and µC frequently exceed the boundaries of Eg, triggering the formation of SEI and cathode electrolyte interphase (CEI), respectively. Here, Eg refers to the energy gap between the lowest unoccupied molecular orbital (LUMO) and the highest occupied molecular orbital (HOMO) of the electrolyte. When µA surpasses the LUMO, spontaneous electron transfer from the anode to the electrolyte induces irreversible reduction reactions, thereby initiating the formation of an unstable SEI layer.^[^ ^1^, ^34^ ^]^

The interfacial stability and electrochemical performance of SMA are critically governed by the composition, structure, and dynamic evolution of the SEI.^[^ ^30^ ^]^ Owing to the high reactivity of Na metal and its strong tendency to lose valence electrons, electron transfer inevitably occurs during the reduction and deposition of Na⁺, leading to the uncontrolled growth of an unstable SEI and triggering a series of parasitic reactions at the interface. As shown in Figure 2b, the SEI typically consists of inorganic compounds (Na_2_CO_3_, NaF, Na_2_O, Na_2_S) and organic salts (ROCO_2_Na, RONa).^[^ ^1^, ^35^, ^36^ ^]^ Ideally, the SEI should possess high ionic conductivity, electronic insulation, chemical stability, and sufficient mechanical robustness to effectively prevent continuous electrolyte decomposition.^[^ ^37^, ^38^ ^]^ However, under practical electrochemical conditions, the heterogeneous composition and dynamic evolution of the SEI often lead to a gradual increase in interfacial resistance. In particular, the local stress generated by Na⁺ transport through the SEI can further compromise its structural integrity (Figure 2c).^[^ ^39^, ^40^ ^]^ The rapid fluctuation of internal interfacial stress induces morphological heterogeneity within the SEI, resulting in local electric field distortion. This, in turn, leads to uneven Na⁺ flux distribution and exacerbates the nucleation and growth of unstable dendrites.^[^ ^41^ ^]^

Moreover, the morphological inhomogeneity of the SEI directly impacts the deposition kinetics of Na^+^. As revealed by the simulation in Figure 2d, on a rough SEI surface, the local deposition rate of Na^+^ varies with the surface topography, where non‐uniform deposition behavior facilitates dendrite nucleation and accelerates their growth.^[^ ^42^, ^43^ ^]^ In addition, repeated SEI rupture and reconstruction during plating/stripping cycles lead to continuous consumption of both active Na and electrolyte, thereby lowering the Coulombic efficiency (CE) of SMBs.^[^ ^44^ ^]^ The situation is further aggravated under elevated temperatures, where intensified interfacial reactions between Na metal and the electrolyte promote parasitic side reactions, deplete electrolyte components, hinder the formation of a stable SEI, and ultimately result in uncontrolled dendrite propagation.

### 2.2 Dendrite Growth

The formation of Na dendrites generally progresses through three distinct stages: i) Film formation stage. During the initial charge–discharge cycles, irreversible reactions occur at the interface between the Na metal and the electrolyte, resulting in the formation of an SEI layer.^[^ ^45^ ^]^ While the SEI partially inhibits further electrolyte decomposition, its structural heterogeneity and instability disrupt uniform Na⁺ transport and promote side reactions.^[^ ^46^ ^]^ ii) Nucleation stage. During the deposition process, Na^+^ is reduced to Na atoms and gradually aggregate to form small nuclei. Due to the surface energy and local electric field fluctuations, the Na deposition itself is not homogeneous (Figure 2e),^[^ ^47^ ^]^ and irregular bumps are formed, which may eventually cover the pristine SEI layer, leading to the premature end of its lifetime (Figure 2f).^[^ ^48^ ^]^ iii) Growth stage. The interfacial electric field distortion will further promote the longitudinal growth rate of the dendrites, resulting in the formation of whisker‐like, mossy or tree‐like typical dendrite morphologies.^[^ ^49^, ^50^, ^51^ ^]^

The persistent growth of Na dendrites poses significant safety risks.^[^ ^52^ ^]^ First, the intensified local current density at dendrite tips accelerates electrolyte decomposition, leading to the depletion of active Na. Moreover, due to the tip effect,^[^ ^51^, ^53^ ^]^ charge accumulation at high‐curvature regions promotes preferential Na⁺ deposition, forming a positive feedback loop that exacerbates dendrite propagation. If not effectively suppressed, Na dendrites may eventually pierce the separator, causing internal short circuits, thermal runaway, and even catastrophic incidents such as fire or explosion. The Sand's time model (Equation 1) offers a kinetic framework to understand the onset of dendritic growth:^[^ ^54^, ^55^ ^]^

(1) $$\tau_{sand}=\pi D\frac{{\left(C_{0}e\right)}^{2}}{{\left(2Jt_{a}\right)}^{2}}$$

This model indicates that within the Sand's time (τ_sand_), the cation concentration at the electrode surface remains nearly depleted, maintaining a dynamic equilibrium between metal ion plating and stripping. However, over time, excess Na^+^ ions accumulate and are reduced, initiating dendritic structures. Therefore, tuning the diffusion coefficient 𝐷, increasing the Na⁺ concentration 𝐶_0_, or reducing the current density 𝐽 can effectively delay dendrite nucleation, thus enhancing battery safety and cycling stability.

### 2.3 Volume Expansion

Due to the lack of structural support, SMA undergo severe volume fluctuations during repeated plating/stripping cycles.^[^ ^56^, ^57^ ^]^ This mechanical instability results in the cracking of the SEI, a drop in CE, and accelerated capacity fading (Figure 2g).^[^ ^58^ ^]^ The fracture of the SEI compromises interfacial stability and leads to the detachment of Na particles from the electrode, forming electrically isolated “dead Na”, which increases interfacial resistance and reduces CE (Figure 2h).^[^ ^34^, ^59^ ^]^ Furthermore, the direct exposure of fresh Na to the electrolyte triggers continuous SEI regeneration. The uneven thickness of the newly formed SEI impedes uniform Na⁺ transport and exacerbates dendritic growth. Simultaneously, ongoing Na exposure accelerates electrolyte consumption, worsens interfacial stability, and deteriorates Na⁺ transport kinetics.^[^ ^60^ ^]^

## 3 Challenges of Existing Commercial Separator Materials

The challenging problems of SMBs, such as Na dendrite growth, unstable SEI formation, significant anode volume expansion, and parasitic reactions between Na metal and electrolytes, place elevated demands on separator design and performance. Although commercially available separators have found application in SIBs systems,^[^ ^61^, ^62^ ^]^ their use in SMBs systems fails to address the key challenges faced by SMBs. In fact, it may even exacerbate the deterioration of Na metal performance, including rapid capacity fading and safety risks associated with battery short circuits. The separator materials currently used in SMBs can be broadly divided into two categories: polyolefin‐based separators and GF separators.

### 3.1 Polyolefin‐Based Separators

Commercial separators such as PP2400 and PP2500 offer the advantages of low cost, mature fabrication techniques, and surface modifiability. However, they exhibit several intrinsic limitations when applied to SMBs:

- **Poor Compatibility with Ester Electrolytes**: The wettability of polyolefin‐based separators to ester‐based electrolytes is poor, often leading to the formation of “dead Na” reduced reversibility of the anode, and accelerated capacity fading.^[^ ^63^ ^]^
- **Insufficient Mechanical/Thermal Propeties**: Moreover, polyolefin separators suffer from limited mechanical strength and thermal stability, making them less effective in suppressing dendrite penetration and prone to thermal shrinkage under elevated temperatures, thereby posing safety concerns.^[^ ^64^ ^]^

### 3.2 GF Separators

The second class comprises GF separators, which are widely adopted in high‐voltage ester‐based electrolyte systems due to their excellent wettability and high electrolyte uptake.^[^ ^20^ ^]^ Nonetheless, GF separators also suffer from application challenges.

- **Excessive thickness and Low Energy Density**: GF separators are typically thick (often >300 µm), which increases cell volume and significantly reduces energy density.^[^ ^65^ ^]^
- **Excessive Electrolyte Consumption**: Their high porosity necessitates large amounts of electrolyte, and since GF themselves do not absorb electrolyte but merely retain it within their pores, electrolyte consumption remains high.^[^ ^66^ ^]^ This in turn intensifies side reactions at the Na‐metal interface and accelerates capacity degradation.
- **Rough Surface**: The random fiber arrangement and surface roughness of GF separators can disrupt the planar interface of the SMA, facilitating dendrite formation. The poor interfacial compatibility between GF and Na metal also leads to the undesired adhesion of deposited Na onto the separator, impairing the uniformity and stability of the SEI and compromising long‐term cycling performance.

In both classes, fundamental structural deficiencies significantly hinder long‐term electrochemical stability in SMBs. As summarized in **Table** **1**, polyolefin separators typically possess pore sizes in the range of ∼40–60 nm, whereas GF separators exhibit larger open structures ranging from 1.2–2.7 µm. These excessively large pores fail to spatially confine Na⁺ flux, resulting in heterogeneous nucleation, uncontrolled dendrite growth, and severe interfacial instability.

**Table 1:** Summary of the Physical and Electrochemical Properties of Conventional Polyolefin Separators (Celgard) and GF Separators (Whatman).

Sample	Porisity	Thickness (µm)	Average pore diameter (µm)	Electrolyte	Electrolyte uptake (wt%)/contact angle (deg)	Ionic conductivity (mS cm^−1^)/Transference number	Cycling (h)	Ref.
PP 2400	41	25	0.043	1 m NaClO_4_ in EC/DEC+5% FEC	80.3%/30	0.49/‐	0.5 mA cm^−2^/0.5 mAh cm^−2^ (Short circuit)	[67]
PP 2400	41	25	0.043	1 m NaPF_6_ in diglyme	–/25	–	–	[67]
PP 2500	55	25	0.064	1 m NaPF_6_ in diglyme	‐/41.66	0.49/0.45	1 mA cm^−2^/1 mAh cm^−2^ (200 h)	[68]
PP 2500	55	25	0.064	1 m NaTFSI in PC/FEC	81.9/80.6	5.9 × 10^−4^/0.21	–	[69]
PE 2730	43	20	NA	1 m NaClO_4_ in EC/DMC/EMC	94/‐	0.16/0.17	–	[70]
PP/PE/PP 2325	39	25	0.034	1 m NaPF_6_ in diglyme	‐/36.1	‐/0.44	1 mA cm^−2^/1 mAh cm^−2^ (500 h)	[71]
GF/A	65.5	260	1.6	1 m NaClO_4_ in EC/DEC+5% FEC	548.9/	6/–	0.5 mA cm^−2^/0.5 mAh cm^−2^ (100 h)	[67, 72]
GF/C	66	260	1.2	1 m NaClO_4_ in EC/PC	360/‐	1.674/0.91	–	[73]
GF/D	66	675	2.7	1 m NaPF_6_ in PC/EC	‐/17.35	0.69/1.209	0.5 mA cm^−2^/0.5 mAh cm^−2^ (80 h)	[74]

Importantly, these vulnerabilities are more detrimental in SMBs than in traditional lithium‐ion systems due to the higher reactivity and lower melting point of sodium metal. The absence of chemical selectivity and physical constraint within the separator matrix allows dendritic filaments to propagate through open structures without mechanically piercing the separator. Particularly in GF separators, dendrites can extend freely through the interconnected voids, leading to short‐circuiting and catastrophic failure. This failure mode is not merely mechanical, but rather a consequence of inadequate control over ionic distribution and Na deposition dynamics. These insights emphasize the urgent need for separator designs that integrate structural regulation, ionic flux homogenization, and interfacial stabilization to effectively suppress dendrite growth and ensure safe, long‐life operation of SMBs.

### 3.3 Emerging Separators

To address the limitations of conventional separators in SMBs, significant research focuses on emerging functional separators, such as cellulose‐based paper separators and electrospun nanowire separators. These advanced materials aim to enhance ionic transport, improve safety, and suppress dendrite growth. For example, Cellulose‐based paper separators offer favorable mechanical flexibility and electrolyte uptake. Their interfacial stability and ionic selectivity can be further enhanced through surface functionalization. Moreover, electrospun separators, composed of highly porous nanofiber networks with superior electrolyte wettability, facilitate uniform Na deposition and help suppress dendrite growth.

However, the practical deployment of these functional separators faces significant constraints:

- Their fabrication processes are often complex and costly.
- Similar to GF separators, they can suffer from non‐uniform pore structure distribution.

Consequently, these factors present barriers to large‐scale implementation and may compromise reliability under real‐world battery operating conditions.

### 3.4 Safety Risk and Regulatory Barriers in Separator Design

In addition to the above material‐specific limitations, there are broader concerns related to the safe and ethical deployment of SMBs using these separators. Many commercially available or emerging separators lack sufficient thermal stability and dendrite suppression capability, which may lead to catastrophic failure modes such as short circuits, thermal runaway, or even fire under abuse conditions. These safety risks are not only technological bottlenecks but also raise ethical concerns when considering real‐world applications in consumer electronics, electric vehicles, and grid storage.

Deploying SMBs without rigorous validation of separator safety and reliability could expose users to unacceptable risks and potentially hinder public trust in next‐generation battery technologies. Therefore, separator design must go beyond electrochemical performance to include intrinsic safety mechanisms, such as flame retardancy, thermal shutdown behavior, and robust mechanical integrity. Moreover, universal safety standards and certification protocols need to be established before widespread adoption can occur.

These challenges highlight the urgent need for holistic separator engineering strategies that integrate materials design, interfacial stability, and system‐level safety considerations. The next sections of this review will explore such strategies and discuss how rational separator architectures can address not only electrochemical demands, but also practical and ethical deployment barriers in real‐world Na battery systems.

## 4 Separator Design Principles for SMBs

As a critical component in Na‐based electrochemical energy storage systems, separators are expected to fulfill three fundamental roles: i) physically isolate the electrodes to prevent short circuits; ii) provide continuous pathways for Na⁺ transport; iii) stabilize electrode–electrolyte interfaces to mitigate parasitic reactions. While both SIBs and SMBs utilize Na⁺ as charge carriers, their fundamentally different anode structures and interfacial chemistries impose distinct requirements on separator design.^[^ ^1^ ^]^

### 4.1 Differences in Separator Requirements Between SIBs and SMBs

**Figure 3:**
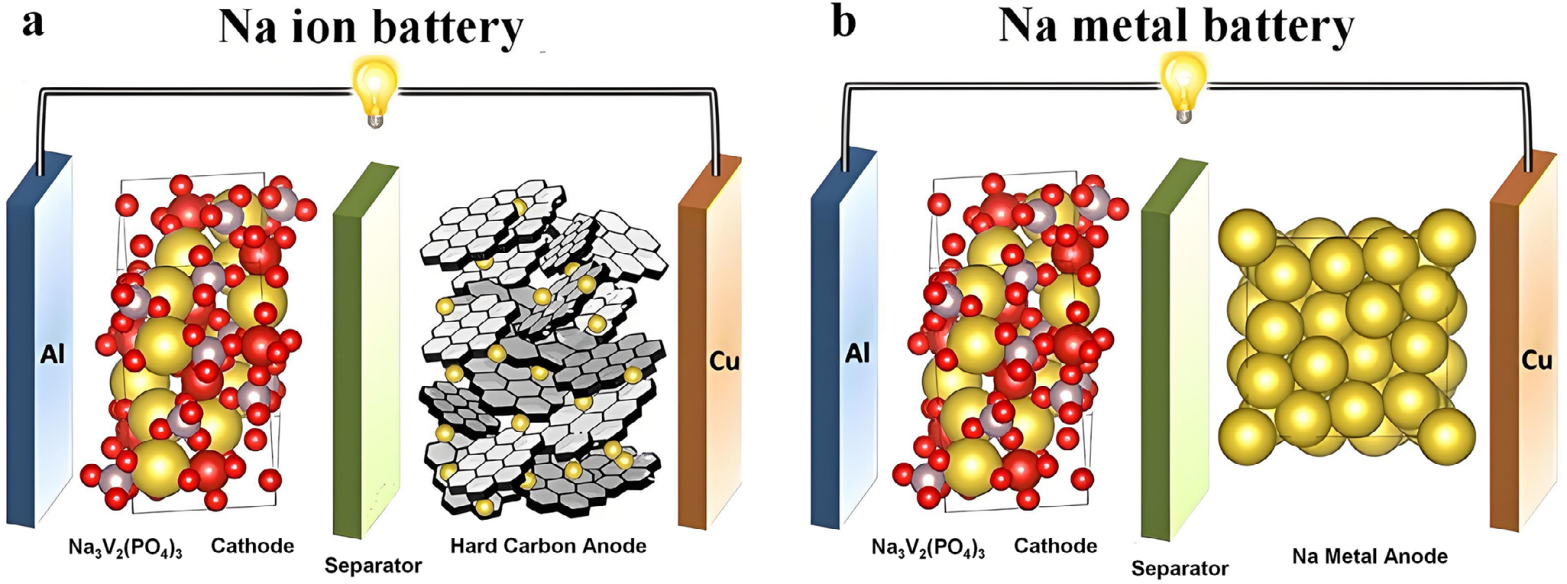
Schematic diagram of different types of Na batteries. a) SIBs, b) SMBs. Reproduced with permission.^[^ ^1^ ^]^ Copyright 2023, The Royal Society of Chemistry.

SIBs operate based on a “rocking‐chair” mechanism (**Figure** **3**a),^[^ ^75^, ^76^ ^]^ in which Na⁺ ions shuttle reversibly between the cathode (e.g., layered transition metal oxides like Na_x_CoO_2_ or Prussian blue analogs such as Na_2_Fe[Fe(CN)_6_]) and the anode (e.g., hard carbon or Na alloys) during charge/discharge processes.^[^ ^66^, ^75^, ^77^, ^78^, ^79^, ^80^ ^]^ During Na⁺ storage, the process predominantly follows an adsorption–intercalation mechanism,^[^ ^81^ ^]^ under which the electrode structure remains relatively stable. Anode materials such as hard carbon and alloys typically undergo minimal volume change during cycling and are inherently free from dendritic metal deposition. The separator in SIBs generally has a high electrolyte uptake to allow efficient Na^+^ ions transport to pass through, excellent chemical and electrochemical stability to withstand the battery environment, and good mechanical strength to prevent the contact between the cathode and the anode. It should also exhibit thermal stability to enhance battery safety and have a porous structure to facilitate electrolyte absorption while preventing dendrite penetration.

By contrast, SMBs utilize highly reactive metallic Na as the anode (Figure 3b), where Na⁺ repeatedly undergoes plating and stripping during charge–discharge cycles. Although a SEI spontaneously forms on the Na surface, it is generally unstable and susceptible to rupture due to volume expansion, uneven current distribution, and the serious parasitic reactions.^[^ ^35^, ^82^ ^]^ Furthermore, different from the hard carbon composed anode of SIBs, the dense and low‐porosity nature of Na anode concentrates Na^+^ ions deposition solely on the electrode surface, while the uneven ion flux distribution further induces the growth of SMBs. Compared to SIBs, ideal SMBs place even higher demands on the separator. In addition to electrolyte uptake, chemical stability, and mechanical strength, the ideal separator of SMBs should have very high electrolyte wettablity and uniform ion flux distribution, which are conductive to the formation of a stable SEI protection layer and effectively suppress the growth of Na dendrites. In SMBs coupled interfaces between the Na metal electrode and conventional separators, a significant structure‐performance relationship exists between electrochemical deposition behavior and the separator's ion transport properties. Traditional separator dominated by micron‐scale porous inert frameworks rely on electrolyte wetting for ion transport. This non‐ion‐conducting separator skeleton inherently causes uneven interfacial ion flux distribution, which not only exacerbates dendrite penetration risks but also triggers inhomogeneous SEI formation. Rational design of the separator's skeleton structure and properties, enabling full‐flux ion transport with uniform flux distribution, is crucial for achieving homogeneous Na metal plating and stripping.

The low hardness of the SMA presents interfacial mechanical stability challenges during cycling. Localized compressive stress exerted by the separator during plating/stripping readily induces microscopic morphological distortion on the anode surface. Under dynamic stress, the SEI layer becomes more prone to fracture and failure, creating a stress‐induced vicious cycle of rupture and reformation. Particularly under high‐capacity testing conditions, the substantial dynamic volume deformation of the SMA significantly exacerbates heterogeneity in the electrode‐separator interfacial stress field distribution. A stable SEI layer is crucial not only for promoting uniform Na metal deposition but also for suppressing parasitic reactions between Na and the electrolyte. Conventional separators, constrained by the ionically inert framework materials, typically adopt large‐pore structures. However, such macroporous separator designs tend to concentrate compressive stress locally. Therefore, in high‐pressure SMBs systems, developing separators that deliver uniform mechanical pressure distribution to the SMA while featuring a smooth and dense surface becomes critically important.

### 4.2 Critical Separator Property Demands for SMBs

**Figure 4:**
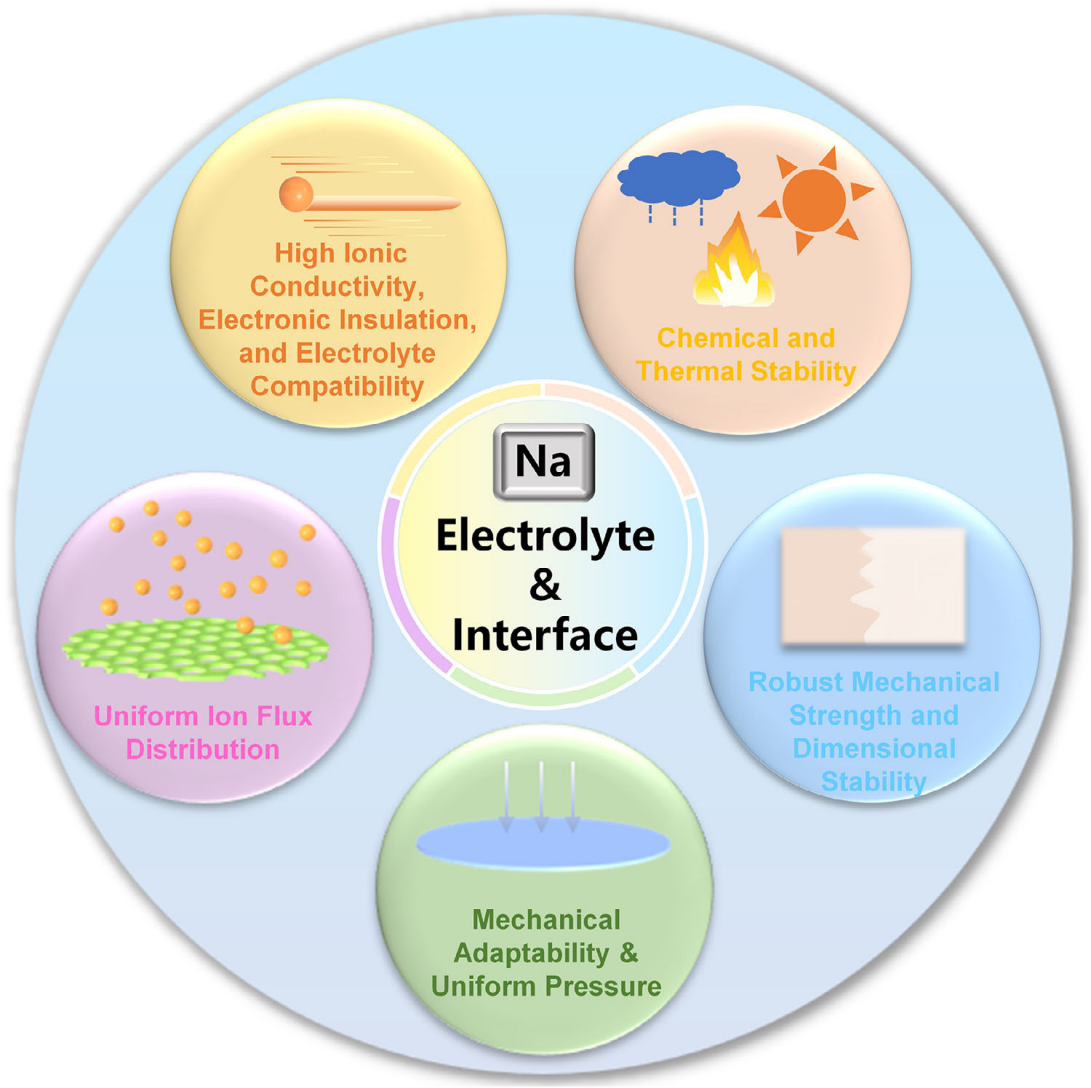
Schematic Design Demands for SMBs Separators.

Separators are no longer regarded as inert physical barriers, but as key functional components that govern ion transport behavior and interfacial stability in SMBs. The high reactivity of Na metal, combined with its tendency for dendritic growth and unstable SEI, imposes stringent requirements on separator design. As illustrated in **Figure** **4**, an ideal separator must concurrently achieve multiple functional targets such as high ionic conductivity, interfacial compatibility, high Na⁺ transference number, uniform ion flux, and mechanical adaptability to realize critical performance outcomes, including stable SEI formation, lowered nucleation barrier, regulated deposition kinetics, and dense, dendrite‐free Na deposition.^[^ ^83^, ^84^ ^]^

#### 4.2.1 High Ionic Conductivity Electronic Insulation, and Electrolyte Compatibility

Achieving high ionic conductivity coupled with an elevated Na⁺ transference number is critical to minimize concentration polarization at electrode interfaces and ensure efficient charge transfer.^[^ ^85^ ^]^ This reduces anion accumulation that exacerbates space‐charge buildup, a key trigger for dendrite nucleation.^[^ ^86^ ^]^ Achieving high ionic conductivity requires separators to exhibit exceptional electrolyte wettability and uptake capacity to ensure complete pore filling and uniform electrolyte distribution, thereby minimizing interfacial resistance. Simultaneously, separators must maintain strict electronic insulation to prevent parasitic electron transfer and self‐discharge.

#### 4.2.2 Uniform Ion Flux Distribution

Homogeneous ion flux distribution across the separator‐electrode interface is essential to suppress localized electric field distortions and “tip effects” that initiate dendritic growth.^[^ ^87^ ^]^ By ensuring spatially uniform Na⁺ supply, preferential deposition at surface protrusions is mitigated, promoting planar plating morphology.^[^ ^88^ ^]^ This is achieved through functional group design, surface charge homogenization, and nanostructure control of separator skeletons to eliminate flux hotspots that destabilize deposition kinetics.

#### 4.2.3 Mechanical Adaptability & Uniform Pressure

Consistent mechanical adaptability is essential to maintain intimate and stable contact between the separator and the dynamically evolving SMA surface during repeated plating/stripping cycles. Traditionally, stress distribution models assume a flat and homogeneous electrode–separator interface, enabling the application of uniform compressive pressure and estimation of idealized stress fields. However, under practical conditions, the Na surface undergoes significant morphological evolution due to dendritic growth, interfacial instability, and local volume fluctuations.^[^ ^89^ ^]^ This results in highly irregular topographies characterized by pits, protrusions, and voids, which lead to heterogeneous stress distributions and localized pressure concentrations. These stress hotspots can induce SEI rupture, degrade interfacial adhesion, and accelerate dendrite intrusion. Compressive stress uniformity remains critical for interrupting the stress‐induced rupture–reformation cycle and preserving SEI integrity over long‐term cycling.^[^ ^90^, ^91^ ^]^ Achieving this requires separators with sufficient mechanical adaptability to conform to nonplanar electrode surfaces while maintaining elastic recovery. This has prompted the development of separators incorporating viscoelastic or elastomeric components that provide both compliance and resilience. Additionally, smooth surface topography engineering is employed to enhance conformal contact and minimize stress concentration. To enable accurate prediction and regulation of interfacial mechanical behavior under realistic battery conditions, future modeling frameworks must consider time‐dependent morphology evolution and chemo‐mechanical coupling at irregular and dynamically changing interfaces.

#### 4.2.4 Chemical and Thermal Stability

To ensure long‐term operational safety and structural integrity in SMBs, separators must exhibit both chemical inertness and thermal resilience. Specifically, excellent chemical stability is required to resist degradation from highly reactive Na metal and electrolyte components. Concurrently, thermal stability, including resistance to shrinkage or deformation at elevated temperatures, is crucial for maintaining dimensional integrity and preventing thermal runaway under abuse conditions. To further enhance interfacial compatibility, surface functionalization strategies, such as the introduction of fluorinated groups, have been employed to facilitate the formation of inorganic‐rich SEI layers, which suppress parasitic reactions and improve interfacial passivation.^[^ ^42^ ^]^ In parallel, the integration of thermally robust ceramic materials has proven effective in enhancing both the thermal durability and the structural rigidity of separators during cycling,^[^ ^92^ ^]^ offering a promising route toward safer and more stable SMBs.

#### 4.2.5 Robust Mechanical Strength and Dimensional Stability

Exceptional mechanical strength and dimensional stability are imperative to withstand puncture forces from Na dendrites and resist deformation during electrode volume fluctuations.^[^ ^64^, ^93^ ^]^ High tensile modulus prevents pore collapse under cycling stress, while minimal thermal shrinkage maintains structural integrity during thermal excursions. Crucially, anisotropic swelling must be suppressed to avoid localized ion flux distortion, or mechanically‐graded architectures that combine rigidity at the Na interface with flexibility in bulk regions.

## 5 Modification Strategies

Conventional PP/PE, GF, and cellulose‐based separators in SMBs suffer from critical limitations such as uneven ion flux, poor interfacial stability, and insufficient capability to suppress dendrite growth. These issues severely compromise the reversibility of SMAs and the overall safety of the battery. To overcome these challenges and achieve the separator design principles introduced above, including uniform ion flux regulation, optimized structural configuration, improved electrolyte wettability, and effective interfacial stress management, researchers have proposed targeted functional strategies. These strategies are based on two main approaches: chemical modification of materials and multiscale structural engineering. The following sections systematically discuss the modification routes and cooperative mechanisms for PP/PE, GF, and emerging functional separators, with a focus on addressing existing material limitations while meeting specific performance requirements.

### 5.1 Optimization of PE/PP Separators

Polyolefin‐based separators, such as polyethylene (PE) and polypropylene (PP), have been widely adopted in commercial battery systems.^[^ ^94^, ^95^ ^]^ However, their intrinsically low polarity and chemical inertness result in poor electrolyte wettability, which easily induces uneven Na deposition, dendrite penetration, and degradation of the SEI in SMBs, thereby severely compromising cycling stability and safety. To address these challenges, recent efforts have focused on an integrated strategy combining interfacial chemical modulation, multiscale structural engineering, and functional material hybridization.

#### 5.1.1 Interfacial Chemical Modulation

**Figure 5:**
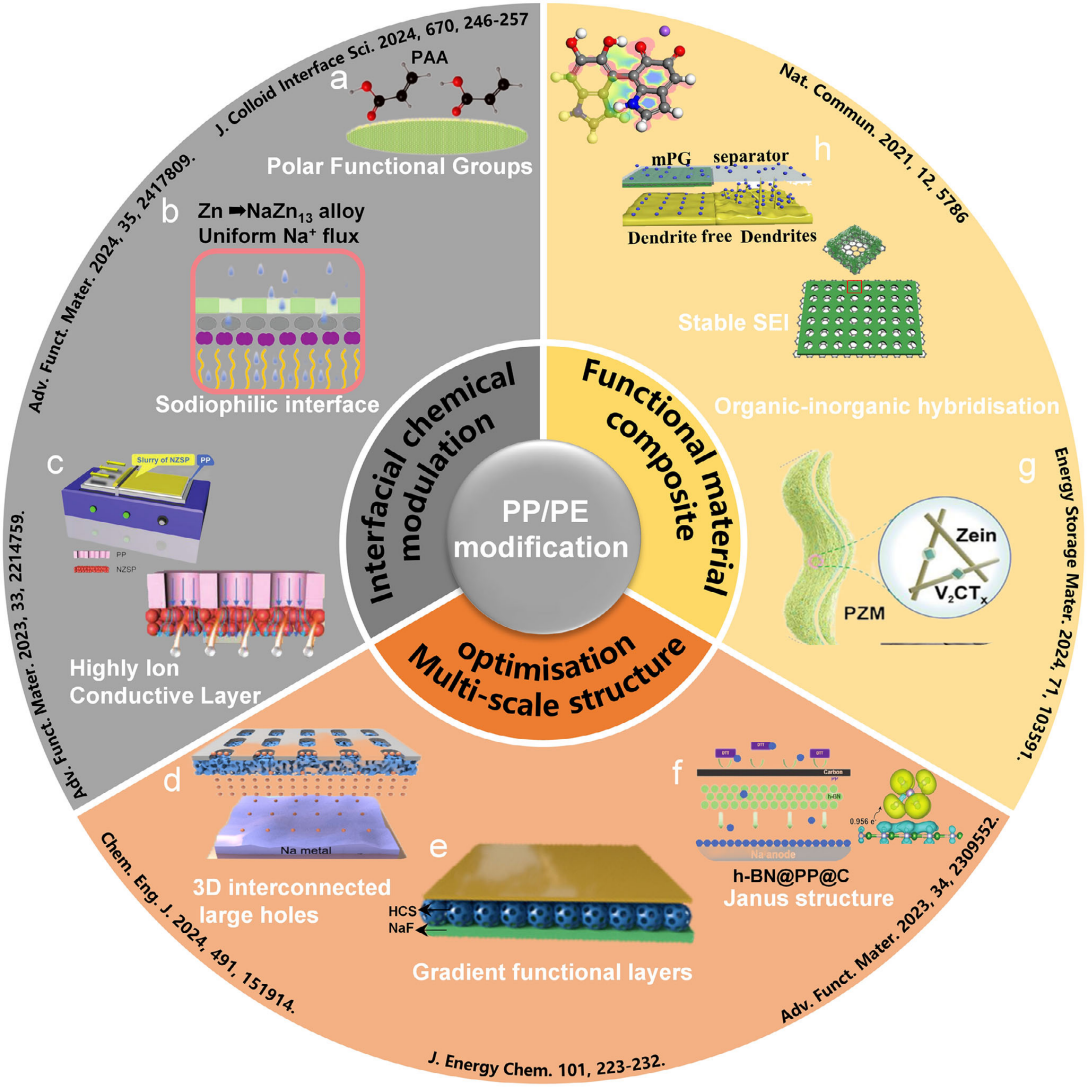
PP/PE modification strategies. a) PP‐g‐PAA separator. Reproduced with permission.^[^ ^71^ ^]^ Copyright 2024, Elsevier. b) Zn‐diamane/PP separator. Reproduced with permission.^[^ ^68^ ^]^ Copyright 2024, Wiley‐VCH. c) NZSP@PP separator. Reproduced with permission.^[^ ^67^ ^]^ Copyright 2023, Wiley‐VCH. d) PP/NH_2_‐diamane separator. Reproduced with permission.^[^ ^98^ ^]^ Copyright 2024, Elsevier. e) PP@HCS‐NaF separator. Reproduced with permission.^[^ ^100^ ^]^ Copyright 2025, Elsevier. f) h‐BN@PP@C separator. Reproduced with permission.^[^ ^102^ ^]^ Copyright 2025, Wiley‐VCH. g) PZM separator. Reproduced with permission.^[^ ^103^ ^]^ Copyright 2024, Elsevier. h) mPG‐coated PP separator.^[^ ^104^ ^]^

Interfacial chemistry regulation plays a pivotal role in enhancing separator wettability and promoting stable SEI formation. The introduction of polar functional groups has emerged as an effective strategy to improve interfacial properties. For instance, electron beam (EB) irradiation has been utilized to graft poly(acrylic acid) (PAA) onto PP separators, introducing carboxyl (─COOH) groups that significantly enhance wettability and Na^+^ ion transference number (t_Na+_ = 0.66), effectively suppressing dendrite growth (**Figure** **5**a).^[^ ^71^ ^]^ This approach is environmentally benign, free of byproducts, and scalable for industrial production.

Constructing sodiophilic interfaces is another critical approach for promoting uniform Na deposition. Wang's group introduced Zn nanoparticles onto the PP separator surface, which lowered the nucleation overpotential to 12 mV and reduced the electrolyte contact angle from 41.66° to 17.21°, significantly enhancing interfacial sodiophilicity and deposition uniformity (Figure 5b).^[^ ^68^ ^]^ Xie et al. deposited sodiophilic tin (Sn) nanoparticles via oblique angle magnetron sputtering, forming an efficient heterogeneous nucleation interface. The shadowing effect during deposition provided buffer space for Na‐Sn alloying, enabling dense and uniform Na deposition. As a result, a high CE of 99.9% was achieved under 3.0 mA cm^−2^/3.0 mAh cm^−2^ over 1200 h.^[^ ^96^ ^]^

The integration of 2D materials further expands the scope of interfacial regulation. Coating hexagonal boron nitride (h‐BN) nanosheets onto PP separators improves both wettability and thermal stability, achieving over 1000 h of stable cycling at 3.0 mA cm^−2^.^[^ ^97^ ^]^ To address the sluggish Na⁺ transport kinetics, constructing ion‐conductive interfacial layers has become a promising direction. Xu et al. introduced a coating of solid‐state electrolyte Na_3_Zr_2_Si_2_PO_12_ (NZSP) onto PP separators, significantly enhancing wettability and ion distribution in carbonate‐based electrolytes, leading to over 1095 cycles in SMB pouch cells (Figure 5c).^[^ ^67^ ^]^

#### 5.1.2 Multiscale Structural Engineering

Multiscale structural design plays a vital role in suppressing dendrite growth and optimizing ion transport. By precisely tuning pore size distribution, constructing 3D interconnected networks, and designing gradient functional layers, uniform ion flux, volume‐change buffering, and enhanced dendrite suppression can be achieved. 3D interconnected architectures effectively enhance mechanical strength and prevent dendrite penetration. For instance, in situ construction of a 3D network of amino‐functionalized 2D nanodiamonds (NH_2_‐diamane) on PP separators increased porosity and Na⁺ transport efficiency while tripling mechanical strength (Figure 5d), enabling stable cycling of symmetric cells at 5 mA cm^−2^ for over 3200 h.^[^ ^98^ ^]^ Gradient functional layers enable spatial separation of functionalities, synergistically optimizing ion migration and dendrite inhibition. In a sandwich‐structured PP‐TiO_2_‐PP separator, the central TiO_2_ layer acts as a mechanical barrier against dendrite intrusion, while the outer PP layers preserve high porosity for efficient ion transport, extending the cycle life of Na‐O_2_ batteries from 82 to 137 cycles at 200 mA g^−1^.^[^ ^99^ ^]^ Further advancements include the PP@HCS‐NaF separator, where the inner nitrogen‐doped mesoporous hollow carbon spheres (HCS) regulate Na⁺ flux, and the outer NaF layer promotes a NaF‐rich SEI (Figure 5e). This design reduces Na⁺ diffusion barriers, achieving 280 stable cycles (CE = 99.6%) in Na||Cu asymmetric cells and ultralow polarization (9 mV) with 6000 h lifespan in Na||Na symmetric cells.^[^ ^100^ ^]^

Janus structure design further deepens spatial functionality separation. Wang et al. developed a Janus separator with a grafted PMTFSINa layer and a nitrogen‐deficient MXene layer, simultaneously tuning anode‐side ion flux and cathode‐side local electric fields.^[^ ^69^ ^]^ This design enhanced the cycling stability and energy efficiency of Na‐S batteries while reducing electrolyte usage by 70%. Similarly, Zheng et al. proposed a MXene@C/PP/MXene@C sandwich structure with asymmetric functional layers for the anode and cathode.^[^ ^101^ ^]^ The cathodic mesoporous carbon shell (1049 m^2^ g^−1^) adsorbs and converts polysulfides, while the anodic MXene@C layer ensures uniform Na⁺ flux. This design enabled RT‐Na‐S batteries to retain 95.8% capacity after 650 cycles at 0.5 C. It is worth noting that anion transport also critically affects interfacial stability. Zhu et al. incorporated positively charged ZIF‐8 frameworks on both sides of PE separators to immobilize free anions, increase the solvent‐separated ion pair (SSIP) ratio, reduce Na⁺ migration energy barriers, and suppress dendrite growth.^[^ ^41^ ^]^ Even with a low N/P ratio of 3, the Na|AAS|NVP cell retained 97.69% capacity after 200 cycles.

Lastly, a carbon‐based h‐BN@PP@C Janus separator demonstrated further potential for synergistic interfacial optimization (Figure 5f).^[^ ^102^ ^]^ The anodic h‐BN coating, with high thermal conductivity and chemical stability, promoted uniform Na⁺ deposition and NaF‐rich SEI formation, while the cathodic carbon layer inhibited shuttle effects from soluble intermediates. This separator enabled Na||Na symmetric cells to stably cycle over 1000 h at 1 mA cm^−2^/1 mAh cm^−2^ and full cells to maintain high capacity and low polarization after 300 cycles at 1 C, showcasing its significant advantages in Na metal interface regulation.

#### 5.1.3 Advanced Composite Strategies

With the deepened understanding of interfacial‐structural synergistic mechanisms, organic–inorganic composite strategies have emerged as a promising route to enhance the performance of polyolefin‐based separators. By incorporating multifunctional components, these strategies enable the simultaneous regulation of wettability, facilitation of ion transport, and enhancement of interfacial stability, thereby laying a material foundation for the efficient operation of SMBs.

For instance, a PAN/Zein‐MXene (PZM) composite separator fabricated via electrospinning combines the mechanical flexibility of the PAN framework with the interfacial modulation capability of MXene nanosheets, delivering excellent ionic selectivity and electrochemical stability (Figure 5g).^[^ ^103^ ^]^ This separator achieves a high Na^+^ transference number (t_Na⁺_ = 0.77) and enables stable cycling over 3500 h at a current density of 1 mA cm^−2^, exhibiting low polarization and outstanding cycling durability. In the realm of heterogeneous composite design, a polydopamine (PDA) and multilayer graphene (mPG)‐based separator significantly boosts performance via dual mechanisms of chemical adsorption and physical confinement (Figure 5h).^[^ ^104^ ^]^ The PDA component, rich in polar functional groups (C═O, ─OH, ‐NH_2_), strongly adsorbs Na⁺ ions, lowers the nucleation energy barrier, and stabilizes the SEI layer. Meanwhile, the 2D mesoporous structure constructed by mPG efficiently buffers volume fluctuations and promotes uniform ion transport, thereby extending battery life and improving overall electrochemical performance. In summary, through tailored interfacial polarity control, microstructural engineering, and multi‐component synergistic design, polyolefin‐based functional separators show immense potential in improving both the safety and performance of SMBs, offering critical support for the development of high‐stability and high‐energy‐density SMBs systems.

### 5.2 Optimization Strategies for GF Separators

As mentioned earlier, conventional polyolefin separators are widely employed in commercial battery systems owing to their low cost and well‐established processing techniques. However, in carbonate‐based electrolytes, they suffer from inherent drawbacks such as poor wettability, limited electrolyte retention, and inadequate thermal stability.^[^ ^105^ ^]^ These issues exacerbate interfacial side reactions and dendrite growth, severely hindering the long‐term stability of SMBs. To overcome these limitations, increasing attention has been devoted to GF separators. Owing to their superior electrolyte wettability, high porosity, and good thermal resilience, GF separators offer a promising platform to construct stable electrode/electrolyte interfaces and suppress dendrite formation. However, challenges such as poor ionic selectivity, low mechanical strength, and interfacial instability hinder their practical application in SMBs. Therefore, systematic optimization strategies are urgently needed to enhance interfacial compatibility, ionic regulation, and mechanical integrity. Recent advances have primarily focused on functional material integration, nanostructure engineering, and bioinspired interface design, as elaborated below.

#### 5.2.1 Functional Material Composites

**Figure 6:**
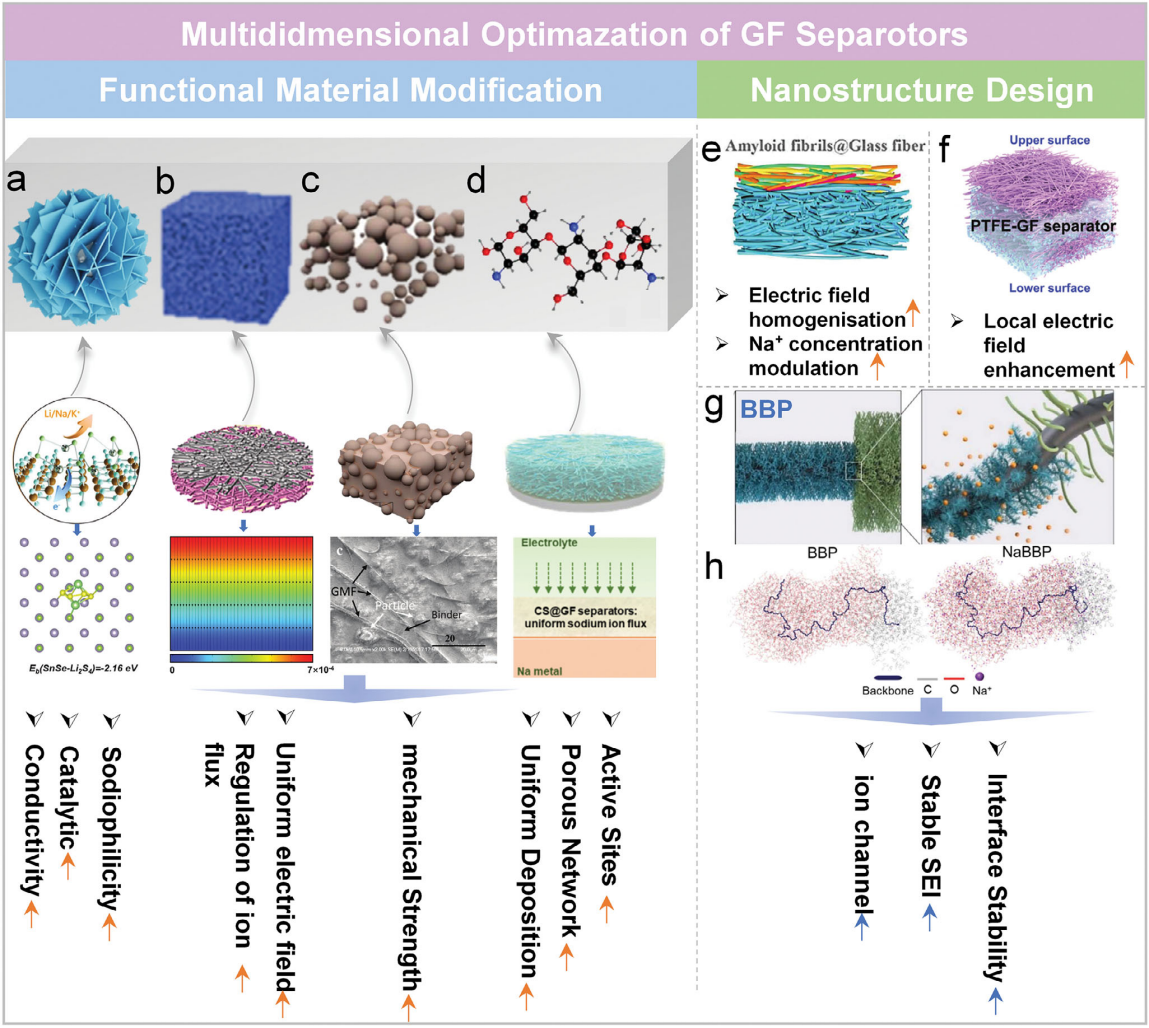
Schematic illustration of strategies for optimizing GF‐based separators, highlighting two main approaches for electrolyte modification: functional material integration and nanostructure design, along with representative examples. a) GF@SnSe separators and relaxed Li_2_S_4_‐adsorbed structure on the surface of SnSe calculated with DFT. Reproduced with permission.^[^ ^106^ ^]^ Copyright 2024, Wiley‐VCH. b) GF@ZIF‐8 separator with simulated current density vectors and electric field distribution. Reproduced with permission.^[^ ^107^ ^]^ Copyright 2025, Wiley‐VCH. c) α‐RGMF after particle injection and corresponding SEM schematic. Reproduced with permission.^[^ ^108^ ^]^ Copyright 2018, Wiley‐VCH. d) CS@GF separator and schematic of uniform Na^+^ flux. Reproduced with permission.^[^ ^86^ ^]^ Copyright 2024, Elsevier. e) Illustration of the AF5@GF separator. Reproduced with permission.^[^ ^111^ ^]^ Copyright 2023, Wiley‐VCH. f) Schematic of PTFE‐GF separators. Reproduced with permission.^[^ ^16^ ^]^ Copyright 2023, Wiley‐VCH. g) Illustration of the GF‐NaBBP separator. h) Optimized molecular dynamics (MD) simulation snapshots of BBP and NaBBP. Reproduced with permission.^[^ ^112^ ^]^ Copyright 2025, The Royal Society of Chemistry.

The introduction of functional materials into GF separators enables synergistic regulation between ion transport, interfacial stabilization, and structural reinforcement. For example, Li et al. introduced SnSe nanosheets with high conductivity and polar adsorption properties into the anodic side of GF separators, and prepared GF@SnSe multifunctional separators (**Figure** **6**a).^[^ ^106^ ^]^ In Na‐S batteries, SnSe facilitated polysulfide adsorption and catalytic conversion, improving rate capability and cycling stability while mitigating shuttle effects. To further regulate ionic flux, Xu et al. functionalized GF separators with ZIF‐8, a porous and negatively charged framework material, to construct GF@ZIF‐8 membranes. This modification not only enhanced puncture resistance but also optimized Na⁺ flux and local electric field distribution, achieving dendrite‐free deposition (Figure 6b).^[^ ^107^ ^]^ As a result, Na||Na symmetric cells delivered a prolonged lifespan of 850 h at high current density, significantly outperforming unmodified GF separators (<180 h), while also exhibiting a higher t_Na⁺_ of 0.48 and outstanding low‐temperature adaptability (78 mAh g^−1^ at −40 °C).

Similarly, Ansari et al. fabricated reinforced glass microfiber (RGMF) composite membranes by embedding high‐modulus inorganic particles (e.g., Al_2_O_3_ or β‐Al_2_O_3_) into the GF matrix (Figure 6c).^[^ ^108^ ^]^ This approach improved structural robustness, dendrite penetration resistance, and gas barrier properties. When applied in Na‐O_2_ batteries, the RGMF separators achieved over 400 cycles with a low charge overpotential (<40 mV) and a high oxygen utilization of 96%.

Carbon‐based functional coatings have also attracted significant attention due to their tunable structures and excellent electrical conductivity. For instance, a composite layer of nitrogen/sulfur co‐doped carbon nanofibers (N,S‐CNF) and carbon black (CB) synergistically stabilized polysulfides in Na─S batteries through chemical adsorption and physical confinement, suppressing active material loss.^[^ ^109^ ^]^ This configuration sustained a reversible capacity of 527 mAh g^−1^ after 900 cycles at 0.5 C.

The integration of polymeric materials has further expanded the functional boundaries of GF separators. Wu et al. developed a poly(ionic liquid)/gel electrolyte hybrid system, which enhanced structural integrity, interfacial wettability, and ionic conductivity, while suppressing side reactions.^[^ ^110^ ^]^ Moreover, Lou et al. utilized chitosan (CS), a natural polymer, to construct a porous CS@GF network. Rich in active sites, this framework facilitated uniform Na⁺ deposition, inhibited electrolyte decomposition and polarization, and significantly improved interfacial stability (Figure 6d).^[^ ^86^ ^]^

#### 5.2.2 Nanostructure Engineering

Nanostructure engineering provides a precise approach for tailoring interfacial behaviors in GF separators. For example, a nanostructured layer based on amyloid fibrils (AF5) was introduced to modulate the local electric field and Na⁺ concentration gradient, promoting the formation of a stable SEI layer composed of Na_3_N and NaN_x_O_y_ (Figure 6e).^[^ ^111^ ^]^ This design extended the lifespan of Na||Na symmetric cells to 1800 h and maintained 87.13% capacity retention over 1000 cycles in Na||NVP full cells. Building on electric field regulation, PTFE nanospheres were further introduced to enhance interfacial electric field intensity, markedly improving both Li⁺ and Na⁺ transport kinetics. As a result, Li||Li and Na||Na symmetric cells achieved stable cycling over 1245 and 2750 h, respectively (Figure 6f).^[^ ^16^ ^]^

Shim et al. developed a bottlebrush block copolymer (BBP) composed of poly(ethylene oxide) (PEO) and polystyrene (PS), which self‐assembled into a hexagonal cylindrical morphology with an entanglement‐free topology (Figure 6g).^[^ ^112^ ^]^ The distinct microphase separation facilitated continuous Na⁺ transport pathways, while the bottlebrush architecture enhanced Na⁺ coordination via the PEO segments, effectively regulating interfacial ion flux and suppressing dendrite growth. MD simulations further visualized the 3D coordination structure of Na⁺ within the BBP framework, revealing uniform ion distribution in the PEO domains (Figure 6h). Thanks to this nanostructure engineering strategy, BBP‐modified GF separators substantially improved electrode/electrolyte interfacial stability and enabled exceptional cycling performance and dendrite suppression even under high‐rate conditions.

### 5.3 Cellulose‐Based Separators

Cellulose, characterized by abundant polar functional groups, excellent mechanical strength, and environmental friendliness, has emerged as a promising separator material for SMBs. However, pristine cellulose membranes suffer from low ionic conductivity and insufficient electrolyte wettability, limiting their ability to effectively suppress dendrite growth. To address these challenges, various functionalization strategies have been developed, including chemical group modification, inorganic/organic composite engineering, conductive polymer integration, and gelation techniques. Importantly, many of these approaches also demonstrate synergistic effects with electrolyte formulations, collectively enabling enhanced interfacial stability and Na⁺ transport.

**Figure 7:**
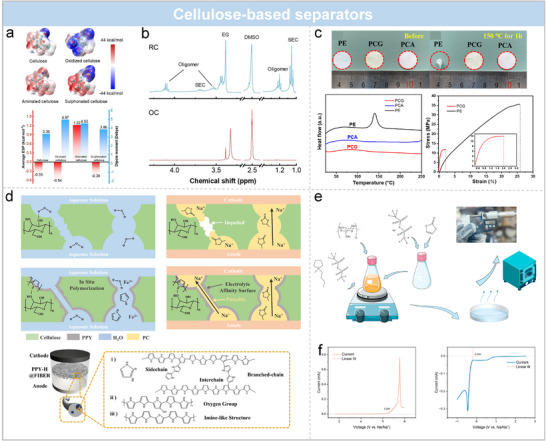
Cellulose separator modification strategy. a) Comparison of ESP distributions and average ESP values and dipole moments for protocellulose, oxidised cellulose, aminated cellulose, and sulphonated cellulose by DFT calculations. b) Organic composition analysis of SEI. Reproduced with permission.^[^ ^17^ ^]^ Copyright 2025, Wiley‐VCH. c) Photographs of PE, PCA, and PCG spacers before and after thermal exposure at 150 °C for 1 h. Comparison of DSC curves of PE, PCA, and PCG spacers and stress‐strain curves of PCG spacers and PE spacers. Reproduced with permission.^[^ ^113^ ^]^ Copyright 2021, Springer Nature. d) Schematic of the modification process and of the polypyrrole‐modified cellulose spacer with structural defects applied to SMBs. Reproduced with permission.^[^ ^114^ ^]^ Copyright 2024, American Chemical Society. e) Preparation of CTA/PyR14TFSI/NaTFSI IGPE membranes. f) Measurement of IGPE ESW. g) LSV curve of stainless steel/IGPE/Na cell with an electrode area of 1.54 cm^2^. Reproduced with permission.^[^ ^115^ ^]^ Copyright 2024, Elsevier.

Introducing polar groups such as carboxyl (─COOH), sulfonic (─SO_3_H), or amino (─NH_2_) moieties into the cellulose backbone can significantly enhance sodiophilicity and optimize the electrolyte–separator interface. Carboxyl‐functionalized cellulose separators promote the cleavage of P─F bonds in NaPF_6_, facilitating the formation of a stable NaF‐rich SEI (**Figure** **7**a).^[^ ^17^ ^]^ Nuclear magnetic resonance analyses confirm that high‐dipole separators inhibit solvent reduction and suppress organic oligomer formation (Figure 7b), leading to lower interfacial resistance and improved mechanical stability. Consequently, Na||NVP full cells achieve over 1000 stable cycles at 1 C with a capacity retention of 94.83%, outperforming batteries with commercial GF separators.

In addition to chemical functionalization, inorganic hybridization has proven effective. Coating graphite‐phase carbon nitride (g‐C_3_N_4_) onto cellulose substrates enhances both ionic conductivity and structural robustness (Figure 7c).^[^ ^113^ ^]^ The self‐supporting layered structure of g‐C_3_N_4_ minimizes binder content, facilitating ion transport and improving cycling performance.

Conductive polymer modification, such as in situ polymerization of polypyrrole (PPy) on cellulose surfaces, offers further enhancements (Figure 7d).^[^ ^114^ ^]^ In this case, the synergistic interaction between the PPy‐modified separator and the electrolyte plays a crucial role in stabilizing the Na interface. The defect‐rich PPy introduces abundant ion pathways, achieving an electrolyte uptake of 254% and a Na^+^ transference number of 0.62. Nitrogen coordination sites selectively promote Na⁺ migration while mitigating electron transport, thereby suppressing dendritic growth. As a result, cells employing this separator demonstrate superior rate performance and more than twofold capacity retention compared to pristine cellulose membranes, even under fluorine‐free electrolyte conditions.

Furthermore, Ma et al. developed a cellulose‐based gel polymer electrolyte (IGPE) utilizing a cellulose triacetate (CTA) scaffold combined with an ionic liquid (PyR₁_4_TFSI) and NaTFSI salt (Figure 7e,f).^[^ ^115^ ^]^ This IGPE demonstrated high ionic conductivity (0.34 mS cm^−1^), a wide electrochemical stability window (5.26 V vs Na/Na⁺), and excellent thermal resistance. Notably, Na||NVP cells with IGPE maintained stable cycling at 60 °C over 100 cycles (0.1 C), highlighting its potential for high‐safety SMBs applications.

### 5.4 Organic Framework‐Based Modifications

Metal‐organic frameworks (MOFs) and covalent organic frameworks (COFs) are crystalline porous materials constructed from metal or organic nodes interconnected by organic linkers.^[^ ^116^ ^]^ Their structural advantages include: i) highly ordered cage‐like architectures with tunable pore sizes enabling selective ion transport; ii) catalytic metal or organic centers enhancing Na ion adsorption; and iii) Sodiophilic functional groups promoting Na⁺ conductivity. Leveraging these characteristics, a variety of MOF‐ and COF‐based separators have been developed for high‐performance SMBs, as discussed below.

#### 5.4.1 MOF Separators

**Figure 8:**
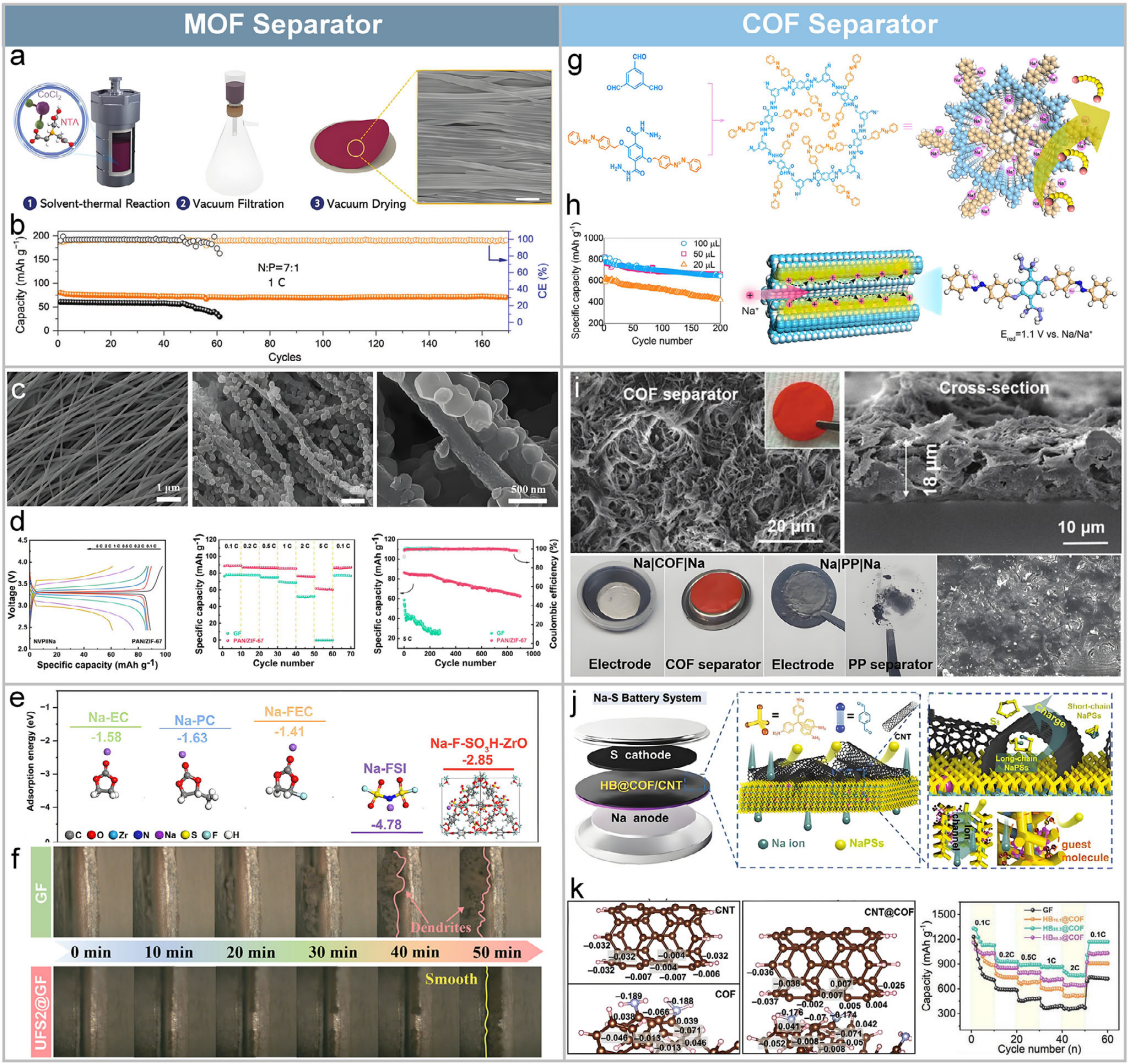
MOF and COF separators modification strategies. a) Schematic of Co‐NWS synthesis and structural characterization. b) Cycling performance of NVP||Na cells using Co‐NWS and GF/D separators at a N/P ratio of 7:1 under 1 C. Reproduced with permission.^[^ ^74^ ^]^ Copyright 2023, Wiley‐VCH. c) SEM images of PAN separator (left) and PAN/ZIF‐67 separator (right). d) Electrochemical performance of NVP||Na cells with various separators. e) Simulated coordination environments and binding energies between UFS2 fragments and NaFSI‐based electrolyte. f) In situ optical images of Na deposition on GF and UFS_2_@GF at different time. Reproduced with permission.^[^ ^118^ ^]^ Copyright 2025, American Chemical Society. g) Synthetic route of azo‐based COFs with Na^+^ selectivity and polysulfide blocking functionality. h) Cycling performance of Azo‐TbTh cells under 1 C with different electrolyte volumes, and corresponding mechanism of fast Na^+^ transport. i) SEM images of the surface and cross‐section of COF separators (top), and post‐cycling images of disassembled Na|COF|Na and Na|PP|Na cells cycled at 20 mA cm^−2^ (bottom).^[^ ^23^ ^]^ j) Proposed mechanism for selective Na^+^ transport and polysulfide rejection using HB/CNT@COF. k) Hirshfeld charge distribution of CNT, COF, and CNT@COF structures. Reproduced with permission.^[^ ^122^ ^]^ Copyright 2023, Wiley‐VCH.

MOF‐based separators have demonstrated significant progress in regulating ion flux, stabilizing electrode/electrolyte interfaces, and suppressing dendrite formation via physical confinement, chemical adsorption, and interfacial modulation. Liu et al. reported a freestanding separator constructed from cobalt‐based MOF nanowire structures (Co‐NWS) (**Figure** **8**a).^[^ ^74^ ^]^ Featuring dense architecture and abundant Sodiophilic polar groups, the separator facilitates uniform Na⁺ deposition and stable SEI formation, rich in NaF and Na_3_N. As a result, it achieves a Na⁺ transference number of 0.83 and ionic conductivity of 1.393 mS cm^−1^. Even under limited electrolyte (10 µL), Na||Cu cells maintained a CE of 98% after 260 cycles, and full cells exhibited over 1500 cycles at 5 C with a capacity retention of 92% (Figure 8b). This work highlights the feasibility of tuning metal node structures to precisely control Na⁺ behavior for advanced separator design.

To further enhance mechanical robustness and functional integration, Hao et al. employed electrospinning to fabricate a PAN/ZIF‐67 composite separator (Figure 8c).^[^ ^117^ ^]^ The Co^2+^ sites in ZIF‐67 selectively adsorb PF^6−^ anions, increasing Na^+^ transference and reducing concentration polarization. Simultaneously, the micro‐mesoporous structure improves electrolyte infiltration and ion transport, while the ordered nanofiber network homogenizes Na^+^ deposition, effectively mitigating dendritic growth (Figure 8d).

Functional group modification offers another effective strategy. Lv et al. introduced fluorine (‐F) and sulfonic acid (‐SO_3_H) groups into a UIO‐66 framework to construct a bifunctional UFS2@GF separator (Figure 8e).^[^ ^118^ ^]^ These groups facilitate Na⁺ desolvation, reduce nucleation overpotentials, and promote uniform dendrite‐free Na deposition. In situ optical microscopy confirmed dense and homogeneous Na deposition, while the SEI layer, enriched with NaF and Na_3_N, enhanced interfacial stability (Figure 8f). Full cells delivered a capacity of 87.3 mAh g^−1^ after 1000 cycles at 10 C, demonstrating the efficacy of multifunctional MOF designs.

#### 5.4.2 COF Separators

COFs are crystalline polymeric materials featuring tunable topologies, high porosity, and excellent thermal stability.^[^ ^119^, ^120^ ^]^ Wang et al. synthesized an azobenzene side‐group‐modified COF membrane with sub‐nanometer pores, which effectively suppressed polysulfide shuttling and reduced ion migration barriers, thereby enabling fast Na⁺ transport and stable interfacial properties (Figure 8g,h).^[^ ^121^ ^]^

To enhance structural durability and current‐carrying capability, Tong et al. introduced sp^2^ carbon‐conjugated frameworks (sp^2^c‐COFs) (Figure 8i).^[^ ^23^ ^]^ These structures exhibited superior mechanical stability and dendrite suppression, maintaining integrity under high current densities (20 mA cm^−2^), outperforming traditional polyolefin separators. Biomimetic designs are also emerging for COF separators. Inspired by cerebrovascular networks, Lu et al. constructed a CNT@COF heterostructure that synergistically regulates selective ion transport and interfacial stability (Figure 8j).^[^ ^122^ ^]^ Density functional theory (DFT) calculations revealed significant charge redistribution at the CNT‐COF interface, promoting electron transfer to polysulfide anchoring sites and accelerating the conversion of long‐chain polysulfides to short‐chain species (Figure 8k). Even under low electrolyte/sulfur ratios, cells with CNT@COF separators demonstrated excellent cycling stability and rate capability, highlighting their practical potential.

### 5.5 Functional Separator Architectures Beyond Conventional Systems

Beyond traditional PP/PE, GF, MOF/COF, and cellulose‐based separators, a variety of emerging separator designs have been developed to address the complex electrochemical and mechanical challenges in SMBs. These advanced systems can be broadly categorized based on their dominant functional mechanisms, including ion sieving, mechanical reinforcement, electrochemical interfacial regulation, and multifunctional heterostructuring, as summarized below.

#### 5.5.1 Ion Sieving and Electrolyte Structure Regulation

Polymer‐based and hybrid separators with tailored surface chemistry and nanostructures have shown promise in selectively guiding ion transport and modulating solvation environments. For example, a nylon 6–cellulose acetate (NCA) composite membrane integrates CONH and OH/ROR/COOR polar groups into a nonwoven structure via electrospinning.^[^ ^123^ ^]^ The resulting dipole–dipole interactions effectively reconfigure the Na^+^ solvation sheath, promoting anion‐rich coordination (C_Na+__anion/C_Na+__solvent ≈0.20) and facilitating the formation of robust, NaF‐rich SEI layers. The membrane exhibits enhanced interfacial homogeneity (137.4 mV), increased ion flux (1.59 mol m^−2^ s^−1^), and dendrite suppression, achieving 96.3% capacity retention after 1600 cycles at 10 C.

**Figure 9:**
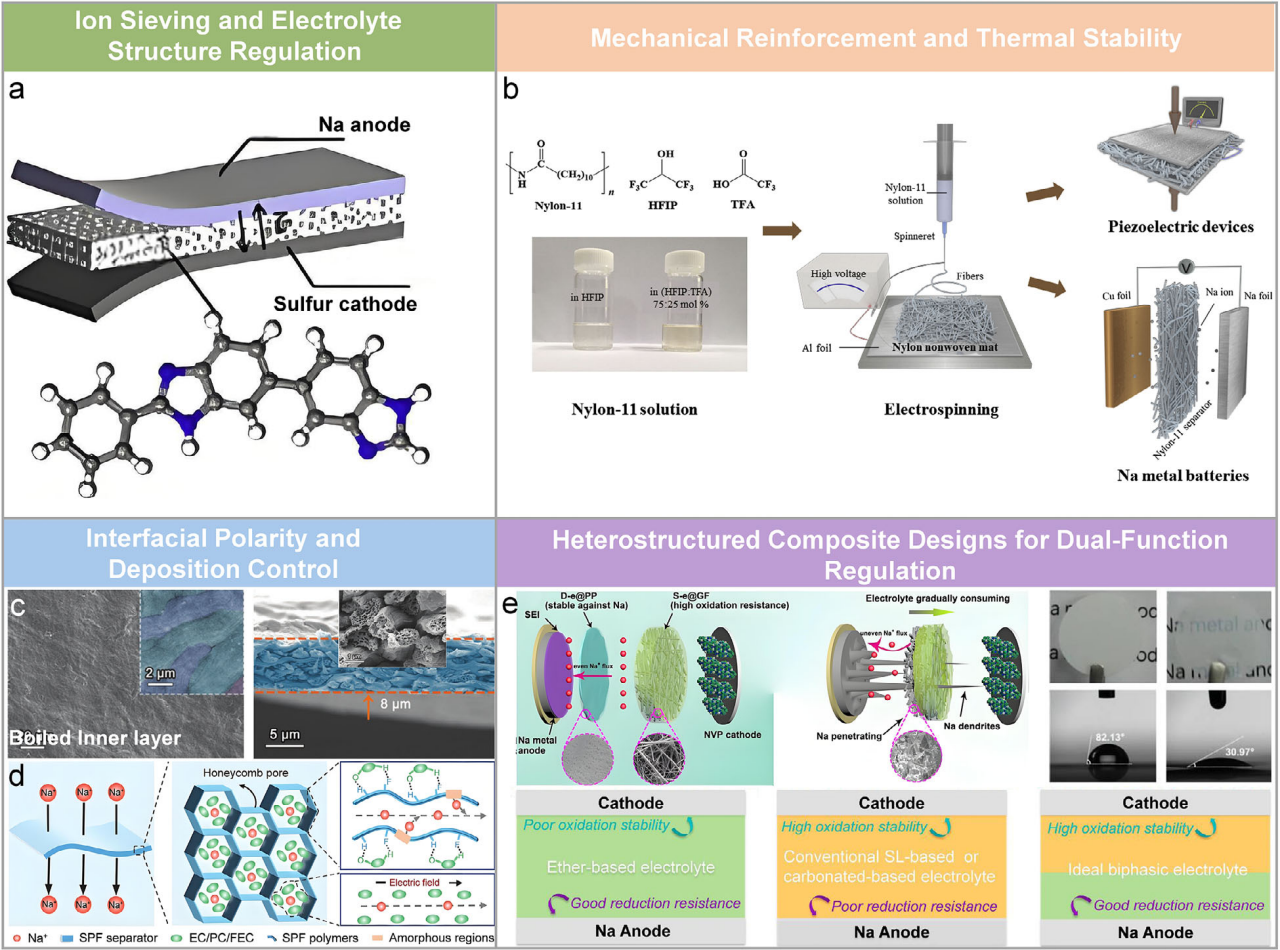
Illustrations of alternative separator types. a) Room‐temperature Na‐S cell configuration and molecular structure of polybenzimidazole (PBI) monomer. Reproduced with permission.^[^ ^124^ ^]^ Copyright 2018, Elsevier. b) Schematic of electrospun Nylon‐11 fiber membrane fabrication. Reproduced with permission.^[^ ^125^ ^]^ Copyright 2020, Elsevier. c) Structure and physical properties of SPF separators, with SEM images of the top surface and cross‐section. d) Schematic of Na^+^ transport through the non‐porous SPF separator. Reproduced with permission.^[^ ^127^ ^]^ Copyright 2023, Wiley‐VCH. e) Effect of different electrolytes on the stability of Na anodes and cathodes. Reproduced with permission.^[^ ^129^ ^]^ Copyright 2022, Elsevier.

Similarly, polybenzimidazole (PBI)‐based membranes demonstrate precise ionic selectivity via their benzimidazole groups, which form N─Na─S coordination bonds with polysulfides in Na─S batteries (**Figure** **9**a).^[^ ^124^ ^]^ Their sub‐10 nm pores enable physical ion sieving, while their high modulus (≈82 MPa) and uniform porous matrix stabilize Na deposition. These properties ensure long‐term cycling in Na||Na symmetric cells without short‐circuiting (100 cycles at 0.25 mA cm^−2^).

#### 5.5.2 Mechanical Reinforcement and Thermal Stability

Electrospun nylon‐11 nanofiber separators, especially those rich in γ‐phase structures, offer superior mechanical strength and thermal endurance compared to commercial PP membranes (Figure 9b).^[^ ^125^ ^]^ The nylon‐H75:T25 separator retains morphology at 150 °C and exhibits twice the electrolyte wettability in NaPF_6_/DME solution relative to Celgard. Its porous architecture facilitates fast Na^+^ transport, boosting rate capability and cycling performance. Furthermore, NaNO_3_@PVDF (NNP91) composite separators incorporate thermally stable, insoluble NaNO_3_ to enhance both mechanical robustness and interfacial properties.^[^ ^126^ ^]^ The NaNO_3_ acts as both a physical filler to reinforce the membrane and a chemical agent that promotes nitride‐rich SEI formation, effectively mitigating dendritic growth and parasitic reactions.

#### 5.5.3 Interfacial Polarity and Deposition Control

Inorganic–organic hybrid and biomimetic membranes offer unique advantages by modulating interfacial polarity to influence deposition behavior. Li et al. introduced a nonporous, honeycomb fibrous membrane based on PVDF‐HFP and PES, where hydrogen bonding interactions create amorphous domains that enhance sodiophilicity and enable uniform Na plating (Figure 9c,d).^[^ ^127^ ^]^ Inspired by nature, a separator derived from silkworm cocoons (SCS) utilizes amino‐rich structures to regulate Na⁺ solvation and promote stable interfacial reactions (Figure 9e).^[^ ^128^ ^]^ This bio‐derived membrane achieves 93.6% capacity retention after 1000 cycles at 10 C, underscoring its practical viability and sustainability.

#### 5.5.4 Heterostructured Composite Designs for Dual‐Function Regulation

To address the conflicting requirements of Na anodes and high‐voltage cathodes, heterostructured membranes with spatially separated functionalities have been explored. A notable design is the D‐e@PP/S‐e@GF bilayer separator, where diglyme‐infused PP and sulfolane‐infused GF are combined to regulate both sides of the cell (Figure 9e).^[^ ^129^ ^]^ The anode‐facing D‐e@PP layer ensures uniform deposition and passivation control, while the cathode‐facing S‐e@GF layer supports high‐voltage compatibility. This architecture enables Na||Na symmetric cells to cycle over 560 times with a CE of 97.22%.

### 5.6 Multidimensional Comparison of Separator Modification Strategies for SMBs

**Figure 10:**
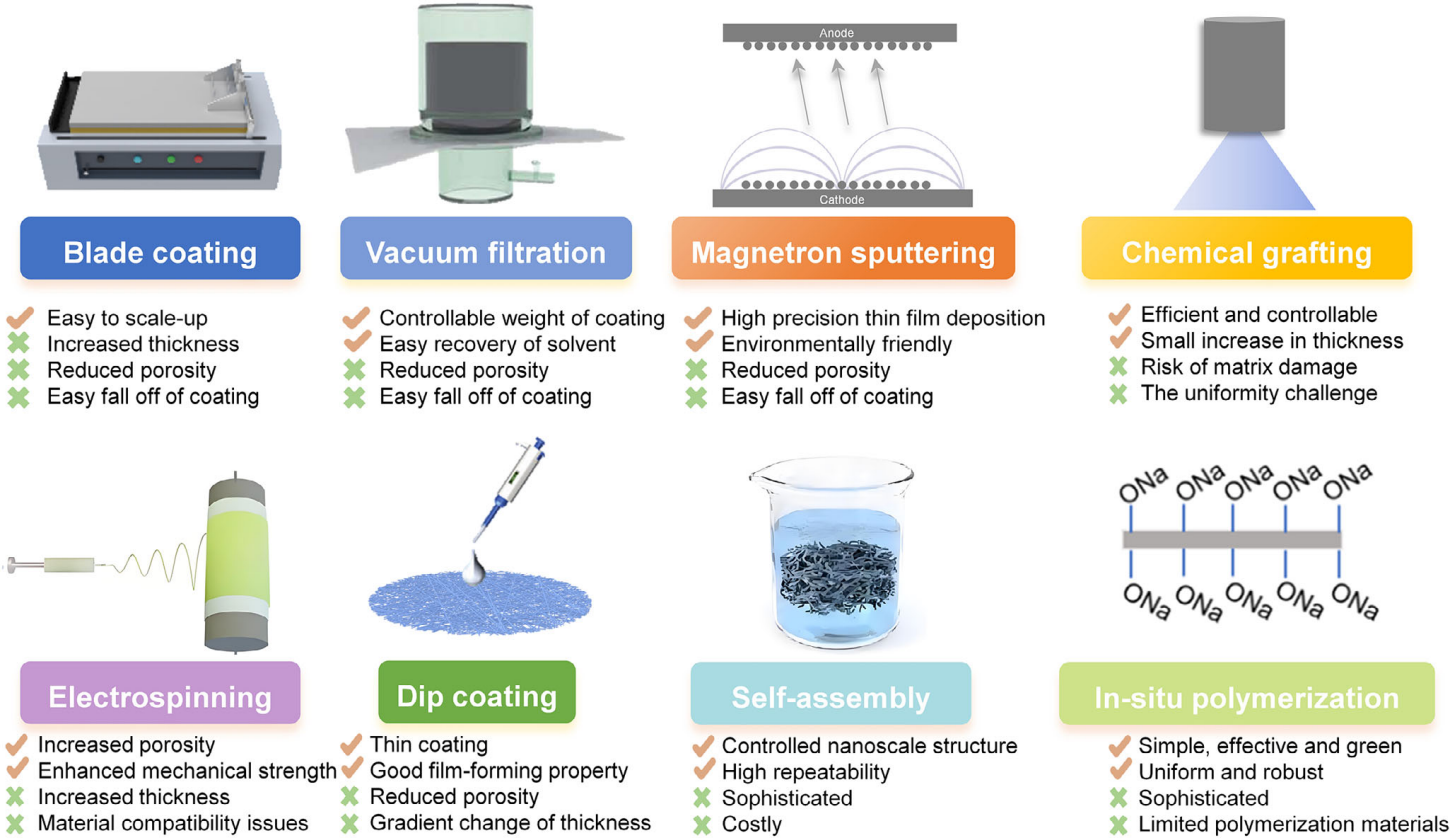
Comparative analysis of separator modification strategies for SMBs.

As illustrated in **Figure** **10**, current separator modification strategies for SMBs can be categorized into eight principal approaches, each exhibiting distinct advantages and trade‐offs in terms of performance and engineering adaptability. Blade coating and vacuum filtration offer excellent scalability and controllable coating uniformity, making them suitable for large‐scale manufacturing; however, insufficient adhesion can lead to delamination or peeling of the functional layer. Magnetron sputtering enables the deposition of uniform and ultrathin functional coatings, offering environmentally friendly processing and high‐precision regulation of ion flux. Chemical grafting allows molecular‐level tuning of interfacial sodiophilicity, rendering it effective for interfacial refinement, though challenges remain regarding coating uniformity and potential damage to the separator substrate. Electrospinning and dip coating can construct high‐porosity nanofiber networks and gradient‐thickness membranes, respectively, effectively balancing mechanical strength and ion transport; however, further efforts are required to improve compatibility between the functional materials and separator matrices. Self‐assembly and in situ polymerization enable multiscale structural construction and interfacial regulation under operational conditions, showing promise for the formation of stable SEI, yet both approaches are constrained by complex synthesis procedures and high production costs. Collectively, each strategy exhibits unique strengths and application scenarios. To accelerate the translation of laboratory‐scale innovations into industrial deployment, it is essential to establish integrated simulation‐experiment frameworks tailored to specific electrolyte compositions, cathode voltage windows, and cycling conditions.

## 6 Effectiveness Evaluation for SMBs Based on Modified Separators

To promote SMBs from laboratory research to practical implementation, establishing a systematic, multidimensional, and standardized framework for separator performance evaluation is essential. While current studies largely focus on cycling stability and interfacial uniformity, there is a lack of consensus regarding key parameters such as evaluation metrics, electrolyte dosage, and the depth of discharge (DOD) for metallic Na. This absence of standardization hinders direct comparisons across different modification strategies. To address this gap, this Review compiles representative electrochemical data from selected literature (**Tables** **2**, **3**, **4**), and correlates separator structure, electrochemical behavior, and full‐cell performance. This structure‐function‐performance mapping provides a conceptual basis for developing rational testing methodologies.

**Table 2:** Overview of modification strategies and performance characteristics of PP/PE‐based separators.

Sample	Thickness (*µm*)	Average pore Diameter (*µm*)	Electrolyte	Electrolyte Uptake (wt%)/contact Angle (deg)	Ionic Conductivity (mS cm^−1^)/Transference Number	Cathode	Electrolyte Content or Weight Ratio of Electrolyte/Active Material	Cyclability [mAh g–1]/ Capacity retention after n cycles	Voltage range (V vs Na^+^/Na)	Ref.
BN‐PP	30	–	1 m NaPF₆ in PC+2% FEC	6	–	–	20 µL	–	−1‐1.5	[97]
PP/NH_2_‐diamane	36	0.15	1 m NaPF₆ in G2	10.3	–	NVP@C	70 µL	87 (150^th^) at 4.27 C		[98]
Zn‐diamane/PP	37	–	1 m NaPF₆ in G2	17.21	1.89/0.66	NVP@C	300 µL	90 (500^th^) at 0.85 C	2.0‐3.8	[68]
Sn‐1.0h‐PP	0.2	–	1 m NaPF₆ in NP‐005	–	–	NVP@C@CNTs	80 µL	89.8 (200^th^) at 2 C	2.5–3.6	[96]
h‐BN@PP@C	47	3‐10	1 m NaPF_6_ in G2	19.8/6.7	0.38	DTT	40 µL	–	0.5‐2.9	[102]
PP‐TiO_2_‐PP	80	–	0.5 m NaSO₃CF₃ in DEGDME	–	–	–	–	–	–	[99]
PP@HCS‐NaF	35	0.5	1 m NaPF₆ in G2	214%/23	2.9 mS cm⁻¹/0.95	NVP	–	70 (300^th^) at 15 C	2.5‐4	[100]
MXene@C/PP/MXene@C	28	0.0045	0.25 m Na_2_S_6_ + 1 m NaCF_3_SO_3_ in TEGDME	21.8	–	S	20 µL	95.8% (650^th^) at 0.5 C	1.5‐3	[101]
NZSP@PP	28	–	1 M NaClO_4_ in EC/DEC+5% FEC	181.4%/6	–	NVP	40 µL	86.5 (1200^th^) at 10 C	2.5‐4	[67]
AAS	–	–	1 m NaPF_6_ in EC/DEC+5% FEC		0.67	NVP	50 µL	91.35% (500^th^) at 0.5 C	2.5‐4	[41]
s‐2D mPG	34	–	1 m NaClO_4_ in PC+5 wt% FEC	8	–	NVP@C	100 µL	100 (500th) at 2 C	2.5‐3.8	[104]
PZM	48	–	1 m NaPF₆ in G2	395/14.2	1.43 mS cm⁻¹/0.77	NVP	–	86.1 (1000th) at 1 C	2‐3.8	[103]
DN‐MXene	28	0.1	1m NaTFSI in the PC:FEC	45.5	0.58	S@C	40 µL	962 (200th) at 0.1 C	0.5‐2.8	[69]
PP‐g‐PAA	–	–	1 m NaPF_6_ in G_2_	240/8.2	0.66	NTP	–	78.51 (350th) at 1 C	1.5‐2.5	[71]

**Figure 11:**
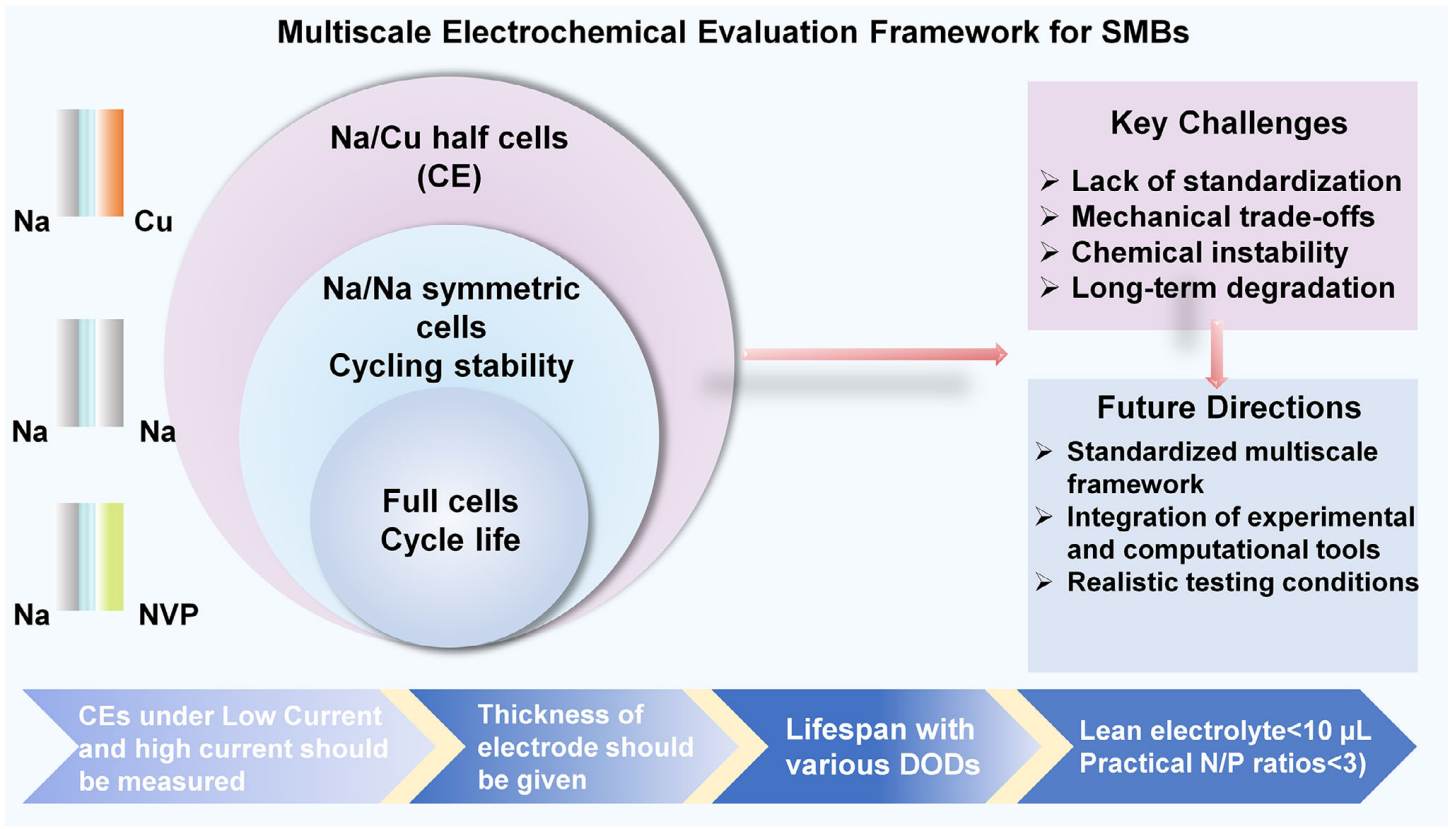
Schematic construction of a multiscale electrochemical evaluation system for SMBs.

Despite growing evidence that separator modification can significantly enhance interfacial stability and electrochemical performance in SMBs, Currently, the absence of consistent testing conditions hinders direct comparison of results across different studies. The development and adoption of standardized electrochemical evaluation protocols is critically important for advancing separator modification research in SMBs. The lack of uniformity obscures genuine performance enhancements attributable to specific modifications, complicates the identification of optimal strategies, and impedes technology transfer from research to commercialization. First, test configurations are inconsistent. Some studies assess CE using Na||Cu (or Al) half cells, others evaluate cycling lifespan with symmetric cells, while a few reports performance in full‐cell configurations, complicating cross‐study comparisons. Second, parameter settings vary widely; current density, electrode thickness, and the electrolyte‐to‐capacity (E/C) ratio are often unspecified or inconsistent. Third, failure mechanism analysis remains limited. Most reports emphasize performance enhancement but offer insufficient insight into SEI composition or dendrite evolution. These challenges indicate the urgent need for a standardized evaluation system encompassing structural parameters, electrochemical metrics, and degradation mechanisms (**Figure** **11**), to enable comparability and accelerate the translation of separator technologies and other modification technologies into scalable applications.

### 6.1 Standardized Electrochemical Evaluation

#### 6.1.1 CE in Na/Cu Half Cells

The CE of Na plating/stripping reflects its reversibility of Na deposition and is typically evaluated in Na/Cu half cells using Equation 2:

(2) $$\begin{array}{c} CE=\frac{stripping\;capacity}{plating\;capacity}=\\ \frac{electric\;charge\;for\;Na^{+}stripping\;from\;active\;metallic\;Na\;\left(mAh\right)}{electric\;charge\;for\;Na^{+}plating+irreversible\;electrochemical\;reaction\;\left(mAh\right)} \end{array}$$

The CE is primarily influenced by side reactions between Na and the electrolyte, while dendrite growth, which results in “dead Na”, also contributes to a decrease in CE. Even under optimized electrolyte systems (such as 1 m NaPF_6_/EC:DMC), maintaining a CE above 99.9% over the long term remains a challenge.^[^ ^132^, ^133^ ^]^ Improving CE relies on effective interfacial regulation strategies that suppress dendrite formation and promote uniform Na deposition. Importantly, CE is highly sensitive to test conditions, particularly current density. At high current densities, SMA encounter aggravated issues due to rapid and uneven Na⁺ nucleation, which enhances local field inhomogeneities and accelerates dendritic growth, increasing the risk of short‐circuit failure. Separator engineering offers critical mitigation pathways under such demanding conditions. Separators featuring vertically aligned nanochannels or ion‐conductive frameworks help homogenize Na⁺ flux and reduce current density hotspots.^[^ ^74^ ^]^ In parallel, mechanically reinforced separators, such as those incorporating ceramic coatings or nanofiber skeletons, provide structural integrity to resist dendrite penetration. Furthermore, surface‐functionalized separators with sodiophilic groups promote uniform nucleation even at high deposition rates. Therefore, separator design plays a pivotal role in maintaining high CE under fast cycling conditions. To fully assess a separator's interfacial regulation capability, systematic multi‐step tests across varying current densities and areal capacities are recommended.^[^ ^134^ ^]^

#### 6.1.2 Cycle Life of Na/Na Symmetric Batteries

Na/Na symmetric cells serve as a simplified model for studying the interface behavior of SMA.^[^ ^135^ ^]^ The cycling stability and polarization behavior of these cells provide insight into the separator's ability to suppress dendrite penetration, alleviate electrolyte depletion, and reduce dead Na accumulation. Ideally, such cells should exhibit low polarization, high stability, and sustain several hundred cycles. However, most studies do not clearly specify the impact of electrode thickness on the DOD, leading to significant discrepancies in DOD under the same capacity setting.^[^ ^136^ ^]^ For instance, at a capacity of 0.5 mAh cm^−2^, an electrode with a thickness of 50 µm corresponds to ≈10% DOD, whereas a thinner electrode can reach up to 50% DOD, causing more pronounced volume changes and interface degradation.^[^ ^54^, ^137^ ^]^ Therefore, it is recommended that studies clearly specify electrode thickness and DOD when reporting cycling life, and integrate morphological evolution analysis to enhance the accuracy and reference value of the evaluation.

#### 6.1.3 Full‐Cell Evaluation under Lean Electrolyte and Low N:P Ratios

Although high CE in half cells and stable cycling in symmetric cells provide a foundation for understanding the separator effect, they are insufficient to fully reflect the practical application potential in SMBs. Na/Cu asymmetric cells and Na/Na symmetric cells typically operate within a low‐voltage range, which does not capture the complex reactions at high‐voltage cathodes. While some studies report performance improvements in full cells with functionalized separators, these are often based on excessive electrolyte use, which may mask failure mechanisms such as dendrite penetration and side reactions.^[^ ^118^ ^]^ Therefore, we recommend adopting “lean electrolyte” testing (E/C ratio < 10 µL mAh^−1^) to better reveal potential issues.^[^ ^74^ ^]^ Additionally, the negative to positive capacity ratio (N:P ratio) should not be overlooked.^[^ ^138^ ^]^ Laboratories often use high N:P ratios (>10:1) to avoid Na depletion, which artificially overestimates energy density and obscures the true capability of separators to suppress dendrites. It is recommended that evaluations be conducted with an N:P ratio ≤3:1 to more closely align with practical applications.^[^ ^139^, ^140^ ^]^ In summary, full cell testing should incorporate high‐voltage cathodes (e.g., NVP or Prussian blue analogs), limited electrolyte, and a reasonable N:P ratio to better reflect real‐world conditions.^[^ ^140^, ^141^, ^142^, ^143^ ^]^ This approach will provide a comprehensive validation of separator modification strategies and lay a solid foundation for the commercialization of SMBs. Establishing universally accepted standards would enable reliable benchmarking, accelerate material optimization, foster collaboration, and ultimately drive the development of high‐performance, commercially viable SMBs by providing objective, comparable data on interfacial stability, cycling stability, rate capability, and safety.

#### 6.1.4 Safety Testing and Performance Validation

Recent studies have shown that separators reinforced with ceramics or nanofibers exhibit enhanced resistance to dendrite penetration.^[^ ^100^, ^117^ ^]^ However, standardized needle penetration test data under sodium‐metal‐specific protocols (e.g., Na||Na pouch cells at defined puncture speeds) are still limited. Future research should adopt or adapt these protocols specifically for SMBs systems. Regarding overcharge performance, certain separators combined with stable electrolytes maintain structural integrity at voltages above 4.5 V versus Na/Na⁺.^[^ ^67^, ^74^ ^]^ Nevertheless, quantitative assessments of thermal runaway thresholds, gas evolution, and dendrite‐induced short circuits during overcharge remain lacking in sodium metal batteries. To address this, incorporating accelerating rate calorimetry (ARC) and real‐time thermal imaging into overcharge testing is recommended to define safer operational limits. Finally, integrating comprehensive safety testing with techno‐economic analysis is crucial for the effective validation and optimization of separators toward industrial application.

### 6.2 Computational Simulations Techniques

#### 6.2.1 DFT Calculations

As the design of separators evolves from structural regulation to mechanism‐driven approaches, computational simulations have become crucial tools for analyzing separator control mechanisms, predicting material behavior, and guiding experimental design.^[^ ^144^, ^145^, ^146^ ^]^ These simulations encompass the entire process from atomic‐level interface interactions to dendrite evolution on a macroscopic scale. At the atomic level, DFT calculations are commonly employed to evaluate the binding energies between Na atoms and functional groups in separator materials, thereby revealing the material's sodiophilicity and anchoring capacity.^[^ ^131^, ^147^ ^]^ Through theoretical calculations, not only can adsorption sites and electronic structure changes be identified, but quantitative guidance for selecting functional groups can also be provided, optimizing the separator's ability to promote Na⁺ transport and inhibit Na dendrite formation.^[^ ^146^ ^]^

#### 6.2.2 Molecular Dynamics Simulations

At the molecular scale, MD simulations offer dynamic insights into ion diffusion behavior in porous separators.^[^ ^148^ ^]^ Previous studies have shown that regularly arranged nano‐channels in COF‐based separators help construct low‐energy, high‐mobility Na ion transport pathways while suppressing solvent‐induced structural expansion. By analyzing MD trajectories, radial distribution functions (RDF), and mean square displacement (MSD), the impact of separator structure on ion diffusion can be quantified, aiding in the selection of porous materials with high selectivity and stability.^[^ ^149^ ^]^

#### 6.2.3 Machine Learning

As separator material design becomes increasingly complex, traditional empirical methods and high‐fidelity simulations face limitations in efficiency and scalability. In recent years, machine learning (ML) has emerged as a powerful tool to accelerate the design of separators for SMBs due to its ability to efficiently handle large material spaces and predict structure‐property relationships. Compared to first‐principles calculations, ML can rapidly extract key performance indicators such as electronic conductivity, phase stability, mechanical strength, chemical compatibility, and ion transport, assisting in the screening and optimization of separator material structures.^[^ ^150^, ^151^, ^152^ ^]^ Several studies have successfully applied ML to predict ion transport performance and chemical stability, with candidate materials validated through simulations and experiments. Nevertheless, the practical application of ML in separator design still faces challenges, including limited datasets, model interpretability, and generalization across different material classes. By incorporating active learning, experimental feedback, and multi‐scale simulations, ML‐guided design is expected to more robustly and efficiently advance the development of next‐generation separator materials.

#### 6.2.4 Phase‐Field and Continuum Models

At the macroscopic level, phase‐field modeling and continuum simulations are widely used to model the growth of dendrites under different structural designs, particularly in multilayer composites, gradient porosity, and asymmetric separator structures. By simulating local electric field distributions, electrochemical reaction interfaces, and ion concentration gradients, the dendrite suppression effect of separators under actual battery operating conditions can be predicted, providing theoretical support for structural design.

Overall, multi‐scale computational simulations not only overcome the time and spatial limitations of experimental characterization but also provide powerful tools for constructing structure‐performance‐mechanism correlations from atomic design to device applications. In the future, combining simulation results with experimental data to develop standardized evaluation models and databases is expected to further accelerate the development of high‐performance SMBs separators.

### 6.3 Characterization Techniques

Separators in SMBs face trade‐offs between structural properties and performance under high‐rate, lean electrolyte, and long‐cycle conditions, with issues such as SEI fragment blockage of pores, delamination of functional coatings, and collapse of layered structures significantly degrading separator performance over time. However, current literature lacks universally accepted solutions addressing these failure mechanisms, and it is important to discuss potential strategies to provide a more comprehensive overview. Recent advances include introducing crosslinked or self‐healing polymer binders, which improve the mechanical integrity of coatings and prevent delamination during cycling‐induced volume changes.^[^ ^114^ ^]^ Furthermore, functional group modifications, including fluorinated or polar groups, promote the formation of stable and compact SEI layers, effectively suppressing fragment detachment and pore blockage.^[^ ^118^ ^]^ These strategies have demonstrated positive effects in prolonging cycle life and maintaining separator performance. To systematically understand and evaluate separator failure and modification effects, a combination of advanced characterization techniques, such as scanning electron microscopy (SEM), in situ transmission electron microscopy (TEM), X‐ray tomography, synchrotron X‐ray imaging, in situ X‐ray diffraction (XRD), operando electrochemical impedance spectroscopy (EIS), Fourier transform infrared spectroscopy (FTIR), and X‐ray photoelectron spectroscopy (XPS), and operando atomic force microscopy (AFM) should be employed to dynamically track microstructure, chemical composition, and interfacial evolution.^[^ ^153^, ^154^, ^155^, ^156^ ^]^ Establishing standardized testing frameworks covering multiple working conditions, scales, and modalities will be key to advancing separator innovation in SMBs.

## 7 Scalable Production and Economic Viability

While advanced separators improve SMB performance, their commercial deployment depends on scalable manufacturing and cost‐effective design. Tables 2, 3, 4 summarize key separator types and electrochemical metrics, providing a basis for evaluating industrial feasibility.

**Table 3:** Overview of modification strategies and performance characteristics of GF‐based separators.

Sample	Thickness (*µm*)	Average pore diameter (*µm*)	Electrolyte	Electrolyte uptake (wt%)/contact angle (deg)	Ionic conductivity (mS cm^−1^)/Transference number	Cathode	Electrolyte content or weight ratio of electrolyte	Cyclability [mAh g^−1^]/ Capacity retention after n cycles	Voltage range (V vs Na^+^/Na)	Ref.
GF@SnSe	270	–	1 m NaClO_4_ in EC:DMC + 5.0% FEC	–	‐/ 0.84	S	40 µL	479 (1000^th^) at 0.6 C	0.8‐2.8	[106]
GF@ZIF‐8	–	0.0017	1 m NaClO_4_ in EC:DMC (1:1) Vol%+5.0% FEC	–	0.98 / 0.48	NVP	–	83 (200^th^) at 1 C	2.5‐3.8	[107]
β‐RGMF	125	0.5‐5	Na triflate (NaTf, 1 m) in ultra‐anhydrous 1,2‐dimethoxyethane (DME) (<1 ppm wate)	–	–	O2	–	87.13% (1000^th^) at 1C	1.6‐3.0	[108]
N,S‐CNF/CB+GF	15 µm (coating)	–	1 mol L^−1^ NaClO_4_ in EC/DEC	–	–	CB/S(71wt%)	–	527 (900^th^) at 0.5 C	0.5‐2.8	[109]
AF5@GF	–	–	1.0 m NaClO_4_ in EC:DEC (1:1 vol%) + 5.0 wt% FEC	–	0.82	NVP	–	–	2.5‐3.8	[111]
CS@GF	–	–	1 m NaPF6 PC: EMC (1:1 Vol%) + 1 Vol% FEC	–	0.46	NVP	120 µL	93.47% (1500^th^) at 5C	2.5‐3.8	[86]
PTFE‐GF	–	–	1 m NaPF_6_ in EC/DEC	–	0.93	NVP	–	91% (340) at 1C	2.5‐3.8	[16]
GF‐NaBBP	–	–	1 m NaPF_6_ in EC:PC:DEC (1:1:1 vol%) + 2 wt% of FEC	451%	–	PBA	–	90% (1000^th^) at 1.7 C	2‐4	[112]
SILGMs	260	1.6	SILGMs	–	0.4	NVP@C	–	99% (100^th^) at 1 C	2.5‐3.8	[110]

**Table 4:** Overview of advanced modification strategies and key performance features of emerging separators.

Sample	Thickness (*µm*)	Average pore diameter (*µm*)	Electrolyte	Electrolyte uptake (wt%)/contact angle (deg)	Ionic conductivity (mS cm^−1^)/Transference number	Cathode	Electrolyte content or weight ratio of electrolyte	Cyclability [mAh g^−1^]/ Capacity retention after n cycles	Voltage range (V vs Na^+^/Na)	Ref.
Cellulose‐based separators										
PCG	46	–	1 m NaClO_4_/EC+PC (1:1, w/w)	258% / 30.7°	1.18 / ‐	NVP	–	115 (100^th^) at 1 C	2.5–4.0	[113]
OC	–	–	1 m NaClO_4_/EC+DEC (1:1, w/w)	262.9% / 17.1°	‐ / 0.82	NVP	–	94.83% (1000^th^) at 1 C	2.5–4.0	[17]
IGPE	60	–	1 m NaTFSI/PyR₁_4_TFSI/VC in EC+PC	–	0.34/0.3	NVP	CTA:PyR₁_4_TFSI:NaTFSI = 1:3:1; 5 wt% VC additive	90% (106^th^) at 0.1 C (60 °C)	2.0–4.0	[115]
PPY‐H@FIBER	–	–	1 m NaClO_4_ in PC (fluorine‐free)	254% / 36.5°	2.77 / 0.62	NVP	–	67.7(100^th^) at 2C	2.0–4.0	[114]
Organic framework‐based separators										
Co‐NWS	75	–	1 m NaPF₆ in PC/EC (1:1) + 10% FEC	–	1.393 / 0.83	NVP	10 µL	92% (1500^th^) at 5 C	2.3–3.9	[74]
PAN/ZIF‐67	146	–	–	425.92% / 0°	1.03 / 0.72	NVP	40 µL	62.5 (800^th^) at 5 C	2.5–3.8	[117]
UFS2@GF	82.5	–	1 m NaClO_4_ EC/PC (v/v, 1:1) + 5 wt % FEC	–	9.306 / 0.67	NVP	75 µL	87.3 (1000^th^) at 10 C	2–4	[118]
Azo‐TbTh COF	0.07	0.00091	1 m NaPF₆ in DME	‐ / 8°	6.9 / 0.89	S/C	–	524(1000^th^)at 1 C	0.5–2.8	[121]
sp^2^‐COF	18	–	1 m NaPF₆ in DIG	–	‐ / 0.78	NTP	–	72%(5000 ^th^)at 10 C	–	[122]
HB/CNT@COF	1.5	–	1 m NaClO_4_ + 0.2 m NaNO_3_ in TEGDME	19.6°	‐ / 0.78	S@C	–	733.4 (400^th^) at 4 C	–	[125]
Other separators										
SCS	230	–	1 m NaClO_4_ in EC/DEC (1:1) + 5% FEC	–	‐ / 0.81	NVP	50 µL	93.6% (1000^th^) at 10 C	2.3–3.9	[124]
SPF	8	–	1 m NaClO_4_ in EC/PC + 5% FEC	complete infiltration	376.7% / ‐	NVP, NaNi₁/₃Fe₁/₃Mn₁/₃O₂ ‐		99.3% (500^th^) at 1 C	3.15–3.38 (NaNi₁/₃Fe₁/₃Mn₁/₃O₂)	[128]
NNP91 (NaNO₃@PVDF)	–	–	1 m NaPF₆ in EC/DEC/FEC	complete infiltration	–	NVP	–	93.1% (4000^th^) at 2 C	2.6–3.8	[127]
γ‐phase nylon‐11	65	–	1 m NaPF₆ in DME	‐ / 0°	–	–	–	–	–	[123]
PBI	–	–	1 m NaTFSI + 0.2 M NaNO_3_ in Tetraglyme	–	–	S@C	100 µL	–	–	[130]
D‐e@PP/S‐e@GF	PP: 25; GF: 300	0.1	D‐e: 1 m NaPF₆ in diglyme; S─e: 1 m NaPF₆ in sulfolane/FEC	61.24°	–	NVP	4 µL on PP; 35 µL on GF	75.3 (3000^th^) at 10 C	2.5–4.3	[131]

Among the separators evaluated, polyolefin‐based separators excel in scalability and cost efficiency. As shown in Table 2, they leverage established lithium battery techniques (e.g., blade coating, roll‐to‐roll processing) for surface modification. Their base material cost is low (≈1.5 USD/kg), though functionalization may add expenses.^[^ ^157^ ^]^ For example, the electrospun PAN/Zein‐MXene separator (PZM) achieves a high Na⁺ transference number (t_Na⁺_ = 0.77) and long cycling stability, but its production involves high energy input and complex equipment, raising concerns about upscaling.^[^ ^103^ ^]^ GF‐based separators, while offering excellent electrolyte wettability and thermal resistance (Table 3), suffer from high thickness and excessive electrolyte demand, which increases cost and reduces energy density. Although reinforced GF@ZIF‐8 or GF@SnSe variants enhance performance and interfacial stability, the raw material cost and manufacturing steps (e.g., solvothermal synthesis, vacuum infiltration) limit large‐scale deployment.^[^ ^106^, ^107^ ^]^ Emerging organic framework‐based separators (Table 4) demonstrate excellent interfacial regulation and dendrite suppression. However, their fabrication often relies on energy‐intensive techniques (e.g., electrospinning, solvothermal growth) and costly precursors (e.g., ZIF‐67 >100 USD/g). These separators also show lower throughput and require strict environmental control, which hinders economic viability despite their excellent performance.

To bridge the gap between performance and processability, future research should emphasize: i) Developing one‐step or solvent‐free coating methods compatible with roll‐to‐roll production; ii) Employing abundant, low‐cost, and eco‐friendly precursors (e.g., cellulose, bio‐derived polymers); iii) Evaluating energy consumption, process yield, and separator cost per Wh at the cell level. These assessments will be essential for translating laboratory‐scale innovations into commercialized SMBs. A standardized techno‐economic analysis framework, integrating data such as ionic conductivity, thickness, transference number, and manufacturing energy input (Tables 2, 3, 4), will provide objective guidance for scalable separator development.

## 8 Conclusion and Perspective

Separator engineering is emerging as a crucial strategy for enabling stable and high‐performance SMBs. This review has provided a comprehensive and mechanistic overview of recent advances in separator design, focusing on dendrite suppression, interfacial stabilization, and ion transport regulation. Modification strategies have been systematically classified across diverse material platforms, including PP/PE‐based, GF‐based, cellulose‐based, and advanced frameworks such as MOFs and COFs, with an emphasis on structure‐function relationships and performance outcomes. To facilitate meaningful performance comparisons, we introduced a multidimensional electrochemical evaluation framework incorporating CE, symmetric cell stability, full‐cell metrics, and safety tests under harsh conditions. Emerging characterization techniques and computational modeling approaches were also discussed for an in‐depth understanding of ion flux distribution, interfacial evolution, and mechanical deformation. Moreover, techno‐economic frameworks evaluating cost, energy input, and process yield are crucial for assessing industrial feasibility and scaling up separator technologies.

Looking forward, the following key directions are expected to play a pivotal role:

- **Uniform Interfacial Contact and Ionic Transport Regulation**: Achieving smooth and homogeneous Na deposition requires both high separator planarity and controlled ion transport pathways. A flat separator surface ensures uniform contact with the SMA, minimizing interfacial resistance and preventing localized current densities that trigger dendrite growth. Simultaneously, controlling pore size distribution, porosity, and structural alignment (e.g., vertically aligned channels) can homogenize Na⁺ flux and reduce concentration gradients. These synergistic improvements at the interface are essential for enabling stable cycling performance.
- **Smart Short‐Circuit Warning Capabilities**: To enhance intrinsic safety, future separators may integrate smart materials or responsive layers capable of detecting early signs of internal failure. By incorporating thermoresponsive polymers or conductive sensing elements, separators can respond to sudden changes in resistance or local temperature spikes, providing real‐time feedback and early warning of potential short‐circuit events, thus mitigating thermal runaway risks.
- **Tailored Architectures and Electrolyte‐Separator Synergy**: Designing separators with advanced architectures such as gradient pores, 3D frameworks, and ion‐selective membranes can significantly improve ion transport efficiency and mechanical robustness. In parallel, co‐engineering separators alongside electrolytes, especially in gel or solid‐state systems, enables better interfacial compatibility and promotes stable SEI formation. Such synergy between structure and chemistry is key to suppressing dendrite penetration and enhancing long‐term cycling stability.
- **Sustainability and Scalable Processing**: Commercial viability requires the use of abundant, biodegradable, and cost‐effective materials. Bio‐based polymers such as cellulose, chitosan, or PLA offer promising sustainable alternatives. Equally important is the adoption of scalable fabrication methods such as electrospinning, slot‐die coating, and roll‐to‐roll processing to transition separator materials from lab‐scale prototypes to industrial manufacturing.
- **Computational Intelligence and Data‐Driven Discovery**: Integrating multiscale computational tools, including DFT, MD, and finite element modeling, can provide detailed insights into the physical and chemical evolution at the separator–electrolyte–electrode interface. Furthermore, ML and high‐throughput screening offer powerful platforms to accelerate material selection, predict separator‐ electrolyte compatibility, and guide structural optimization. These tools can bridge experimental innovation with system‐level predictive design.
- **Temperature‐Responsive Design and Extreme‐Condition Stability**: Temperature significantly influences Na nucleation and plating behaviors. At high temperatures (HT), sodium exhibits higher reactivity and kinetic activity, while at low temperatures (LT), reduced ion mobility leads to uneven deposition and dendrite formation. Separators must therefore be engineered to maintain structural integrity and ionic conductivity across a wide thermal range. Future designs should emphasize thermal resilience (e.g., shrinkage resistance ≥150 °C) and low‐temperature performance (e.g., ionic conductivity at −20 °C or below). Advanced characterization techniques such as in situ TEM, operando X‐ray tomography, and temperature‐resolved impedance spectroscopy are vital for real‐time monitoring of interfacial dynamics under such conditions. A deeper understanding of these thermal effects will pave the way for robust, temperature‐resilient separators suited for real‐world SMBs deployment.
- **Interdisciplinary Strategies Driving Innovative Separator Design**: Inspired by natural systems and empowered by advanced manufacturing technologies, the design of battery separators is increasingly evolving through interdisciplinary integration. Biomimetic architectures, such as those mimicking plant phloem or capillary networks in animals, offer structural blueprints for directional ion transport. Simultaneously, state‐of‐the‐art fabrication techniques, including atomic layer deposition, 3D printing, and microfluidic templating, enable precise control over separator architectures across multiple length scales. Promoting collaboration among materials science, chemical engineering, and computational modeling disciplines holds great promise for addressing complex trade‐offs under multi‐objective optimization constraints and for establishing application‐oriented, system‐level design frameworks.

In summary, through the integration of interfacial engineering, structural design, sustainable materials, and computational intelligence, separators are expected to evolve into multifunctional, intelligent, and scalable components. The synergistic advancement of nanofabrication, predictive modeling, and interdisciplinary innovation will bridge fundamental research and real‐world deployment. In the long term, separator engineering will play a central role in advancing high‐safety, high‐energy‐density, and commercially viable SMBs.

## Conflict of Interest

The authors declare no conflict of interest.

## Data Availability

The data that support the findings of this study are available from the corresponding author upon reasonable request.
